# Increases in 4‐Acetaminobutyric Acid Generated by Phosphomevalonate Kinase Suppress CD8^+^ T Cell Activation and Allow Tumor Immune Escape

**DOI:** 10.1002/advs.202403629

**Published:** 2024-09-26

**Authors:** Xinyi Zhou, Zhiqiang Chen, Yijiang Yu, Mengjiao Li, Yu Cao, Edward V. Prochownik, Youjun Li

**Affiliations:** ^1^ Department of Colorectal and Anal Surgery Zhongnan Hospital of Wuhan University Hubei Key Laboratory of Cell Homeostasis College of Life Sciences Frontier Science Center for Immunology and Metabolism Medical Research Institute TaiKang Center for Life and Medical Sciences Wuhan University Wuhan 430071 China; ^2^ Division of Hematology/Oncology Children's Hospital of Pittsburgh of UPMC The Department of Microbiology and Molecular Genetics The Pittsburgh Liver Research Center and The Hillman Cancer Center of UPMC The University of Pittsburgh Medical Center Pittsburgh Pennsylvania 15224 USA

**Keywords:** 4‐Acetaminobutyric acids, GABAA receptors, immune escapes, PMVK, tumor metabolisms

## Abstract

Certain metabolites in the tumor microenvironment (TME) can alter innate immunity. Here, it is shown how phosphomevalonate kinase (PMVK) allows hepatocellular carcinoma (HCC) cells to overcome the anti‐tumor immunity mediated by CD8^+^ T cells. In HCCs, depletion of PMVK is required to facilitate CD8^+^ T cell activation and their subsequent suppression of tumor growth. Mechanistically, PMVK phosphorylates and stabilizes glutamate decarboxylase 1 (GAD1), thus increasing the synthesis of γ‐aminobutyric acid (GABA), which normally functions as a neurotransmitter. However, PMVK also recruits acetyl‐CoA acetyltransferase 1 (ACAT1) and allows it to convert GABA, to 4‐acetaminobutyric acid (4‐Ac‐GABA), which is released into the TME. There, 4‐Ac‐GABA activates the GABAA receptor (GABAAR) on CD8^+^ T cells, which inhibits AKT1 signaling. This in turn suppresses CD8^+^ T cell activation, intratumoral infiltration, and the anti‐tumor response. Inhibiting PMVK or GABAAR in HCC mouse models overcomes resistance to anti‐PD‐1 immune checkpoint therapy. These findings reveal non‐canonical and cooperative functions among the key metabolic enzymes PMVK, GAD1, and ACAT1 that reprogram glutamine metabolism to synthesize a potent CD8^+^ T cell inhibitor 4‐Ac‐GABA. Blocking 4‐Ac‐GABA signaling in CD8^+^ T cells, particularly when combined with immune checkpoint inhibition, potentially represents a new and potent form of immunotherapy.

## Graphical abstract

PMVK phosphorylates and stabilizes GAD1 to increase GABA synthesis. ACAT1 is recruited by PMVK and converts GABA to 4‐Ac‐GABA, which is released into the TME. 4‐Ac‐GABA activates GABAAR on CD8^+^ T cells to inhibit AKT1 signaling, CD8^+^ T cell activation, and anti‐tumor response. Blocking 4‐Ac‐GABA signaling in CD8^+^ T cells, combined with ICB, potentially represents a novel form of immunotherapy.

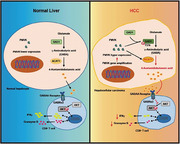

## 1 Introduction

HCC is one of the deadliest and most prevalent cancers worldwide.^[^ ^1^ ^]^ HCC incidence is affected by many factors including pre‐existing diseases such as alcoholic and non‐alcoholic cirrhosis; metabolic factors and environmental and dietary toxins.^[^ ^2^ ^]^ Although the current treatment of HCC mainly includes surgical resection, tumor ablation, and chemotherapy, long‐term survival remains dismal.^[^ ^3^ ^]^ Recently immunotherapy has revolutionized the treatment of some cancers.^[^ ^4^ ^]^ However, only 20% HCC patients benefit from immune checkpoint blockade (ICB) therapy. A major reason for this sub‐optimal response is that tumors often remodel their tumor microenvironment in ways that allow non‐lymphoid immune cells to contribute to T cell inactivation and exhaustion.^[^ ^5^ ^]^ Thus, the immune microenvironment and distinct etiology‐dependent immune features play crucial roles in the response to ICB‐based therapies.^[^ ^3^, ^6^ ^]^ Chronic hepatitis B infection drives the expression of inhibitory immune checkpoint proteins while hepatitis C infection can cause the accumulation of depleted and dysfunctional CD8^+^ T cells.^[^ ^2d^ ^]^ Nonalcoholic steatohepatitis is often associated with diffuse inflammatory infiltration and a complex network of immune cell‐liver cell interactions has been found to facilitate immune escape.^[^ ^2b^ ^]^ Checkpoint molecules are gateways for anti‐tumor T‐cell responses. The targeted therapy of immune checkpoint receptors PD‐1 and CTLA‐4 is the focus of immunotherapy for solid tumors.^[^ ^6^ ^]^ PD‐1 and PD‐L1 interaction lead to extensive dephosphorylation of T cell activation kinase and thus T cells inactivation.^[^ ^5b^ ^]^ CTLA‐4 suppresses the interaction of B7 ligands with CD28, thereby decreasing the abundance of activated CD4 and CD8 T cells.^[^ ^6^ ^]^ The immune checkpoints that inhibit the activity of T cells also include TIM‐3 and LAG3.^[^ ^6b^ ^]^ These and other immune checkpoints have become the focus of extensive clinical research.^[^ ^6b^ ^]^ However, our understanding of the molecular underpinnings governing immune responses and evasion remains incomplete.

Metabolic reprogramming of the TME has an important impact on the occurrence and progression of tumors and can significantly alter tumor immune sensitivities.^[^ ^7^ ^]^ Tumor metabolic remodeling may also lead to the accumulation of additional immunomodulatory metabolites in the TME.^[^ ^8^ ^]^ These metabolites may play non‐metabolic roles by affecting the development and activity of T‐cell immunity.^[^ ^7^, ^8^, ^9^ ^]^ Therefore, studying the metabolic cross‐talk between tumor and immune cells in the TME and linking tumor‐intrinsic metabolism with immune evasion mechanisms and immunotherapy may provide novel therapeutic insights.

PMVK catalyzes the cation‐dependent reaction between Mevalonic‐5‐phosphate and ATP to form mevalonate‐5‐diphosphate and ADP and represents a key step in the mevalonate pathway for isoprenoid/sterol biosynthesis and protein prenylation.^[^ ^10^ ^]^ Although the mevalonate pathway has been implicated in various aspects of cancer development and progression,^[^ ^10^, ^11^ ^]^ the role(s) of PMVK in the establishment of tumor immunity by altering tumor metabolism remains unknown.

Many types of cancer are addicted to glutamine, which contributes to both metabolism and energy supply.^[^ ^12^ ^]^ Glutamine can be converted into GABA by glutamate decarboxylase (GAD) in neurons, which acts as an inhibitory neurotransmitter and reduces the excitability of neurons in the mammalian central nervous system, thus producing a calming effect.^[^ ^13^ ^]^ GABA has been previously studied as a neurotransmitter and a sedative although other studies have pointed to other functions. For example, GABA can regulate the growth, metastasis, and anti‐tumor immune response of cancer cells by shaping the TME in solid tumors such as colon cancer^[^ ^14^ ^]^ and breast cancer.^[^ ^15^ ^]^ GABA, secreted by tumor cells into the TME, can inhibit GSK‐3β activity by activating GABAB receptors on the surface of tumor cells, enhancing β‐catenin signal transduction, and then stimulating tumor cell proliferation.^[^ ^16^ ^]^ Activated B cells also synthesize and secrete GABA to curb CD8^+^ cell immunity against colon cancer.^[^ ^9^ ^]^ Moreover, GABA can be converted to a variety of derivatives, but their roles in cancer and cancer immunity are worth studying.^[^ ^17^ ^]^

Here, we report a hydrophilic metabolomic analysis of murine primary HCCs that revealed elevated levels of 4‐Ac‐GABA, a metabolite of the above‐mentioned GABA derivatives. Mechanistically, PMVK was found to stabilize GAD1 by phosphorylating its C‐terminal threonine at position 576, leading to the reprogramming of glutamine metabolism that favored the synthesis of GABA. At the same time, PMVK was shown to interact with and stabilize ACAT1, thereby allowing it to acetylate GABA and further increase the level of 4‐Ac‐GABA. In turn, 4‐Ac‐GABA suppressed CD8^+^ T cell activation and infiltration and abrogated the antitumor effects of these cells. Finally, a combination of PMVK inhibitor and an anti‐PD‐1 antibody displayed marked therapeutic efficacy against HCC.

## 2 Results

### 2.1 PMVK Expression Correlates with HCC Immune Escape

**Figure 1:**
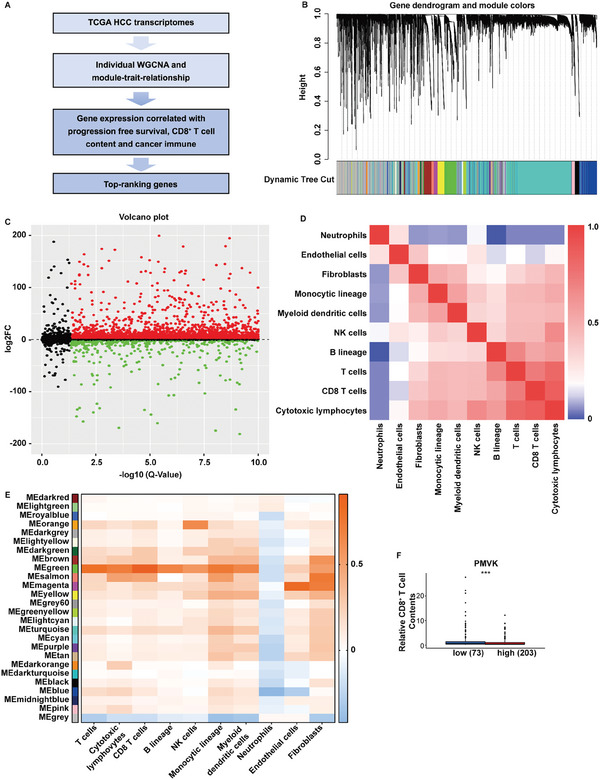
PMVK expression correlates with HCC immune escape by WGCNA. A) The Process of WGCNA screening. B) Expression network analysis revealed 26 different gene modules, representing genes with similar expression changes in samples. C) Volcano plot of DEGs between HCC tissues and paired adjacent tissues analyzed by limma package. D) MCP counter package and ssGSEA methods were used to perform immune infiltration analysis of the gene expression matrix of tumor tissue to obtain heat map of immune correlation. E) The samples were clustered according to the MCP counter score results, and the blue module was positively correlated with CD8^+^ T cells. F) Tumor‐infiltrating CD8^+^ T cell content analysis was performed based on the expression of PMVK in HCC tissues.

To explore the HCC immune‐related changes in the gene expression patterns of patient‐derived HCC, weighted correlation network analysis (WGCNA) was performed on whole RNA‐seq data from 369 HCC tissues and 50 paired adjacent normal tissues from the TCGA‐LIHC database (**Figure** **1**A). The resulting co‐expression network revealed 26 distinct gene modules representing genes with similar expression changes across all samples (Figure 1B). Differentially expressed genes (DEGs) between HCC tissues and their paired adjacent tissues were analyzed using the limma package (Figure 1C). Immune infiltration analysis was performed on the gene expression matrix of tumor tissue using the MCP (Microenvironment Cell Population) counter package and ssGSEA method, and immune correlations were obtained (Figure 1D). Based on the MCP counter‐scoring results, the samples were clustered and classified into high and low immune response groups. The blue and pink modules were positively correlated with CD8^+^ T cells and the high immune group, respectively (Figure 1E). This was most significant for subsequent analysis through analyzing the correlation between gene modules and MCP counter results. In addition, to the correlations between gene modules and MCP counter‐immune grouping, the pink module was positively correlated with the high immune group (Figure 1E). Overlapping these two sets of module genes with DEGs revealed a total of 268 genes (Intersect 1) (Table S1, Supporting Information). Based on the CD8^+^ T cell content results of the MCP counter, the samples were divided into high and low CD8^+^ T cell content groups, and differentially expressed genes were analyzed using limma. Overlapping these differentially expressed genes with Intersect 1 resulted in 36 genes (Intersect 2). Interestingly, there are a total of 18 of these 36 genes whose expression correlated with progression‐free survival (Table S1, Supporting Information, Extended Data Figure 1A,B). Reverse specificity analyses of the expression of each gene associated with tumor‐infiltrating CD8^+^ T cell content, which can indicate a predilection for immune escape, identified 7 of these 18 genes (PMVK, ATP6V1F, IER2, MRPL51, PSMB6, SF3B5 and TRIR), with PMVK being the only gene that was negatively correlated with tumor‐infiltrating CD8^+^ T cell content (Figure 1F, Extended Data Figure 1C and Table S1, Supporting Information) and thus deserving of further investigation.

### 2.2 PMVK Activity Negatively Correlates with CD8^+^ T Cell Infiltration and HCC Immune Escape

**Figure 2:**
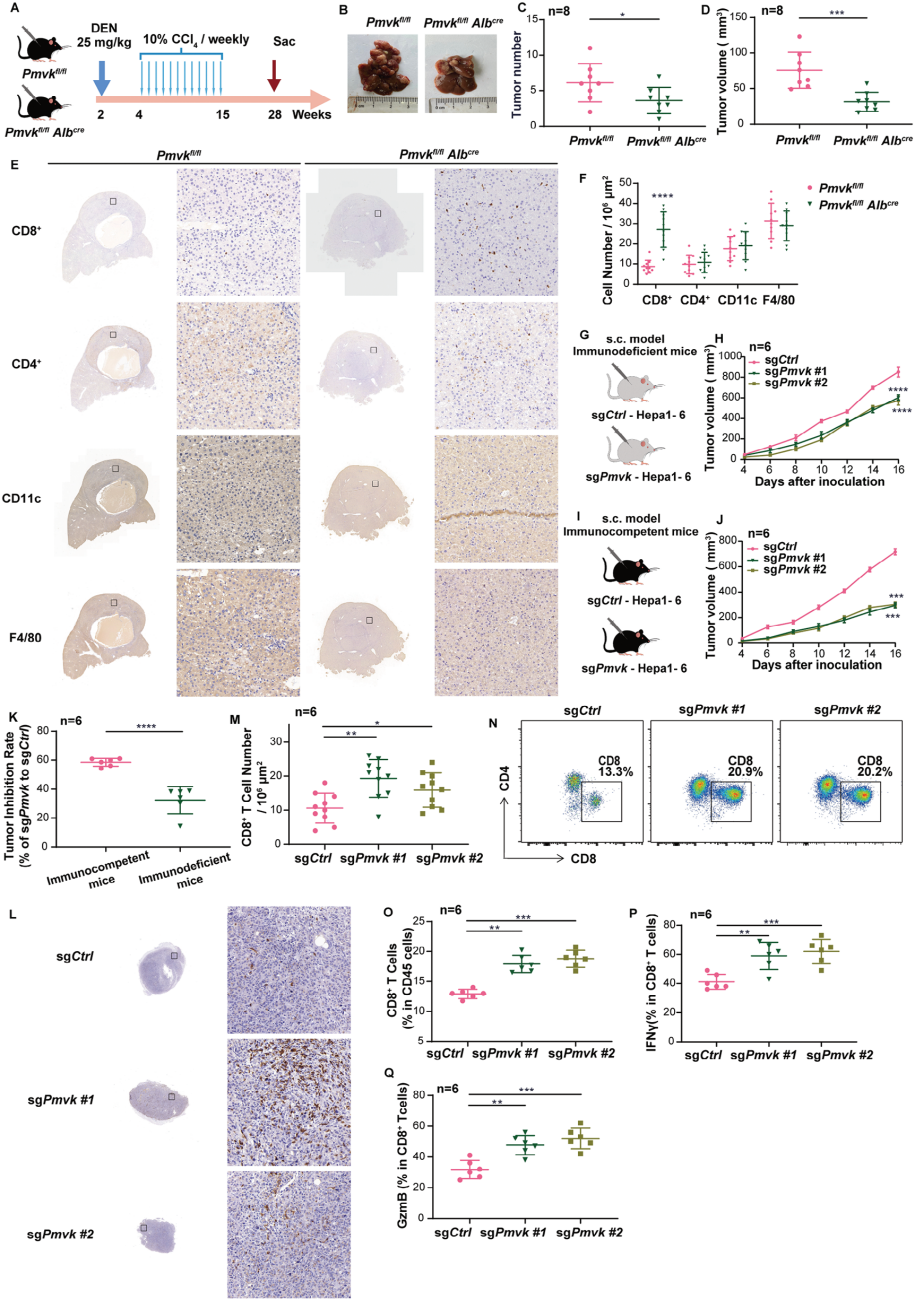
PMVK deficiency enhances CD8^+^ T cell infiltration and activation to inhibit tumor immune escape. A) Schematic overview of DEN/CCl_4_ induced HCC model. *n = *8 mice per group. B) Liver images obtained from the indicated mice from (A). *n = *8 mice per group. C) Tumor numbers in each liver from (A). D) Average tumor volume from each liver from (A). E) Representative immunohistochemical staining of CD8^+^, CD4^+^, CD11c, and F4/80 from A. In each group of samples, the same sample was sliced and stained with different labels. Scale bar, 100 µm. The immunohistochemical staining experiment was performed twice. F) Cell number in 10^6^ µm^2^ area image from (E). *n = *10 images per group. G,H) Subcutaneous xenograft experiments were performed with immunodeficient mice injected with Hepa1‐6 cells stably expressing the indicated plasmids. *n = *6 mice per group. I,J) Subcutaneous xenograft experiments were performed with immunocompetent mice injected with Hepa1‐6 cells stably expressing the indicated plasmids. *n = *6 mice per group. K) Subcutaneous xenograft growth in immunodeficient mice and in immunocompetent mice. *n = *6 mice per group. L) Representative immunohistochemical staining of CD8^+^ T cells from (I). Scale bar, 100 µm. The immunohistochemical staining experiment was performed twice. M) Cell number in 10^6^ µm^2^ area image from (L). *n = *10 images per group. N) Pictures of flow cytometry gating of CD8^+^ T cells in subcutaneous xenograft from (J). Cells were stained as described in Materials and Methods and doublets were excluded using FSC‐A and FSC‐W. Upper panels show percentages of CD8^+^ T cells of live CD45^+^ T cells. O) Flow cytometry shows percentages of CD8^+^ CD45^+^ T cells from (N). *n = *6 mice per group. P) percentages of CD8^+^ T cells expressing IFNγ in subcutaneous xenografts from (J). *n = *6 mice per group. Q) percentages of CD8^+^ T cells expressing GzmB in subcutaneous xenograft from (J). *n = *6 mice per group. Data are shown as mean ± SD.

To further determine the regulatory role(s) of PMVK in HCC immune cell infiltration, PMVK hepatocyte‐specific conditional knockout (CKO) mice were constructed and tumors were generated using the DEN/CCl_4_ model (**Figure** **2**A). CKO mice had significantly reduced tumor numbers and volume compared with control WT mice (Figure 2B–D). Immunohistochemical staining also showed that the infiltration of CD8^+^ T cells in CKO HCCs was significantly increased (Figure 2E,F).

In another model, a PMVK‐deficient Hepa1‐6 mouse hepatoma cell line was constructed and transplanted into immunocompetent C57BL/6J mice and immunodeficient BalB/c nude mice. The inhibitory effect of PMVK loss on tumor growth in immunocompetent C57BL/6J mice was more obvious than that in immunodeficient BalB/c nude mice (Figure 2G–K, Extended Data Figure 2A–D). Both immunohistochemical staining and flow cytometric analysis also showed that CD8^+^ T cell infiltration was significantly increased in *Pmvk‐*deleted HCCs (Figure 2L–O). Further flow analysis revealed additional changes in the TME‐associated CD8^+^ T cell population from *Pmvk*‐deleted HCCs that included increases in the levels of IFNγ and granzyme B (GzmB) (Figure 2P,Q, Extended Data Figure 2E) without significant changes in the levels of PD‐1, TIM‐3 and CTLA‐4 (Extended Data Figure 2F–J). Furthermore, depletion of CD8^+^ T cells significantly promoted the growth of transplanted tumors. CD8^+^ T cell depletion can partially restore tumor growth inhibition induced by knockdown PMVK (Extended Data Figure 2K–M). These data suggested that the effects of PMVK knockout on HCC growth are indirect and involve the infiltration and activation of CD8^+^ T cells. Further analysis showed that PMVK was mainly enriched in tumor cells of HCC tissues (Extended Data Figure 2N).^[^ ^18^ ^]^

### 2.3 PMVK Modifies GAD1 and Interacts with ACAT1 to Increase 4‐acetylaminobutyric Acid Levels

**Figure 3:**
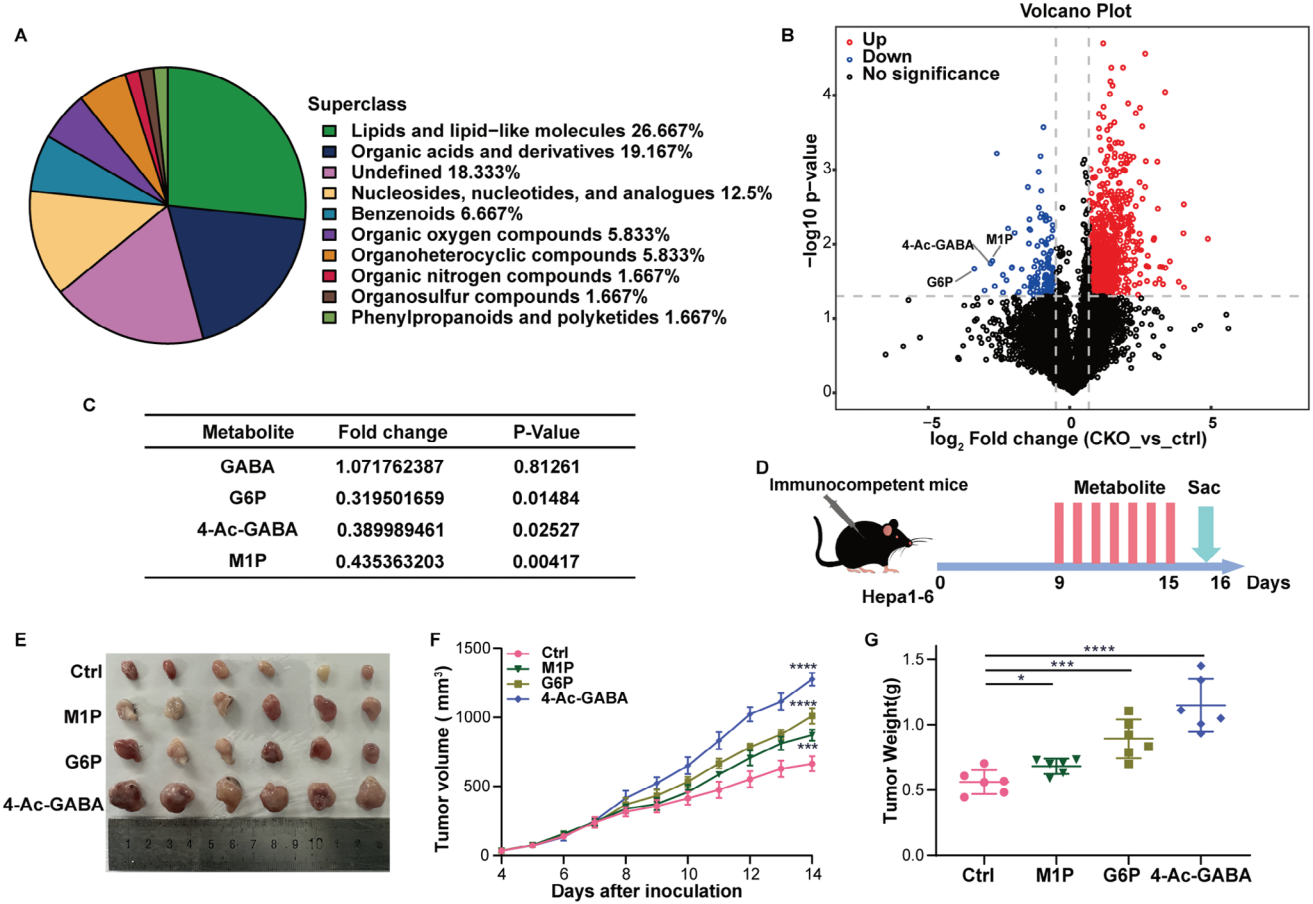
Hydrophilic metabolomics analysis reveals elevated 4‐Ac‐GABA in HCC tissues. A) Cluster plots of hydrophilic metabolomics analyses performed on DEN/CCl_4_ induced HCC tissues from PMVK hepatocyte‐specific CKO mice and WT mice. B) Volcano plot showing metabolite screening distribution. Blue dots indicate significant deletion of metabolites (negative selection). C) Fold change and P‐value of down‐regulated metabolites. G6P, Glucose‐6‐phosphate. 4‐Ac‐GABA, 4‐Acetaminobutyric acid. M1A, Mannose‐1‐phosphate. D) Subcutaneous Hepa1‐6 xenograft experiments in immunocompetent mice with the indicated treatment. M1P, G6P, and 4‐Ac‐GABA were administered intraperitoneally. *n = *6 mice per group. E) Xenograft images from the indicated mice from (D). F) Subcutaneous xenograft tumor volume from (E). *n = *6 mice per group. G) Subcutaneous xenograft tumor weight from (D). *n = *6 mice per group. Data are shown as mean ± SD.

To explore the mechanism(s) by which PMVK levels in tumor cells impacted CD8^+^ T cells, hydrophilic metabolomics analyses of PMVK‐CKO and wild type (WT) HCCs were performed (**Figure** **3**A,B and Table S2, Supporting Information). The results showed that the levels of 4‐Ac‐GABA, glucose‐6‐phosphate (G6P), and mannose‐1‐phosphate (M1P) were significantly down‐regulated in PMVK‐CKO tissues while the level of GABA remained unchanged (Figure 3C and Table S2, Supporting Information). To explore the functional relevance of these metabolic differences, mice with transplanted murine HCCs were treated with these metabolites (Figure 3D). Interestingly, 4‐Ac‐GABA treatment significantly promoted tumor growth relative to other metabolites (Figure 3E–G).

**Figure 4:**
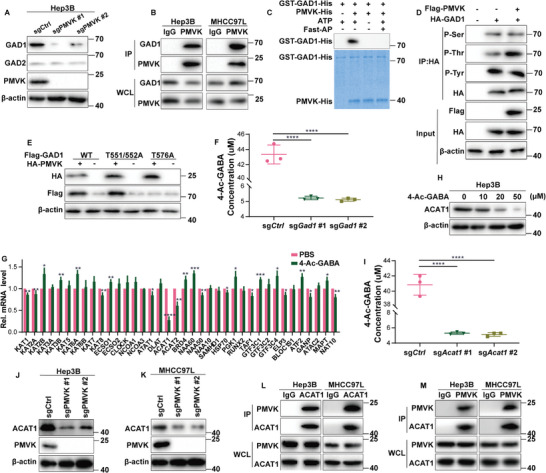
PMVK recruits GAD1 and ACAT1 and increases 4‐acetylaminobutyric acid levels. A) GAD1, GAD2, and PMVK protein expression in Hep3B cell lines. B) Endogenous interaction between PMVK and GAD1 in Hep3B and MHCC97L cell lines. Mouse Anti‐PMVK antibody was used for IP. Mouse IgG was used as a negative control. WCL, whole cell lysate. C) Recombinant purified GST‐GAD1‐His and PMVK‐His proteins were used for in vitro kinase reactions. Fast AP, Fast alkaline phosphatase. D) PMVK phosphorylates GAD1 at threonine residue(s). Immunoprecipitation of HA‐GAD1 with anti‐HA‐magnetic beads followed by immunoblotting with the indicated antibodies. The p‐Ser, p‐Thr, and p‐Tyr denote pan phospho‐serine, phospho‐threonine, and phospho‐tyrosine antibodies, respectively. E) HEK293 cells were transfected with vectors expressing Flag‐GAD1 or its mutants and HA‐PMVK. Cells were collected 48 h later and examined with the indicated antibodies. β‐actin was used as the loading control. F) The levels of 4‐Ac‐GABA in GAD1 knockout Hepa1‐6 cell lines, as measured by UPLC‐MS/MS. *n = *3 samples per group. G) qRT‐PCR analysis of various acetylase genes in murine splenic CD8^+^ T cells with or without 4‐Ac‐GABA treatment, *n = * 3. Data are shown as mean ± SD. H) Hep3B cell lines were treated with 4‐Ac‐GABA at different times and examined with the indicated antibodies. β‐actin was used as the loading control. I) The levels of 4‐Ac‐GABA in ACAT1 knockout Hepa1‐6 cell lines, as measured by UPLC‐MS/MS. *n = *3 samples per group. J,K) ACAT1 and PMVK protein expression in Hep3B cell lines and MHCC97L cell lines. L,M) Interaction between endogenous PMVK and ACAT1 in Hep3B and MHCC97L cell lines. Anti‐PMVK antibody from the mouse as an IP antibody. Mouse IgG was used as a negative control. WCL, whole cell lysate.

4‐Ac‐GABA is the downstream product of N‐terminal acetylation of GABA^[^ ^19^ ^]^ and GAD1 and GAD2 have been reported to be the key metabolic enzymes for GABA (Extended Data Figure 3A).^[^ ^15^ ^]^ While GAD1 and GAD2 transcript levels were unchanged in PMVK‐KO tumors (Extended Data Figure 3B) GAD1 protein level was significantly down‐regulated (**Figure** **4**A, Extended Data Figure 3C). Co‐immunoprecipitation (Co‐IP) experiments of endogenous proteins indicated a direct interaction between PMVK and GAD1 (Figure 4B, Extended Data Figure 3D). Phosphorylation of GAD1 by PMVK was further confirmed by in vitro phosphorylation experiments (Figure 4C). Phosphorylation of GAD1 threonine was associated with PMVK as confirmed by pan‐phosphorylation immunoprecipitation (Figure 4D). Further fragment analysis revealed that the C‐terminal of GAD1 interacted with PMVK (Extended Data Figure 3E,F). When the C‐terminal threonine 576 residue was mutated to alanine, no phosphorylation of PMVK was observed (Figure 4E). The half‐life of GAD1 was also found to be significantly prolonged when Thr 576 was mutated to contain the phospho‐mimetic aspartate residue (Extended Data Figure 3G). GST pull‐down assay also proves the same result (Extended Data Figure 3H). Together, these data suggest that PMVK phosphorylates GAD1 at threonine 576 and increases its stability. Finally, knockout of Gad1 in Hepa1‐6 cells significantly reduced 4‐Ac‐GABA levels (Figure 4F), thus indicating that GAD1's post‐translational regulation by PMVK is correlated with 4‐Ac‐GABA levels.

While the above studies have shown that PMVK phosphorylates GAD1 at Thr576, and increases its stability and the accumulation of GABA, precisely how 4‐Ac‐GABA is produced and whether PMVK is involved in this process remain unclear. To explore this question, Hep3B cells were treated with 4‐Ac‐GABA, and the mRNA levels of various acetylases were measured. ACAT1 transcripts were found to be significantly reduced (Figure 4G), thus suggesting that ACAT1 may be involved in acetylating GABA. 4‐Ac‐GABA also significantly downregulated the protein expression of ACAT1 in HCC cells (Figure 4H, Extended Data Figure 3I). The protein concentration of ACAT1 was correlated with the duration of 4‐Ac‐GABA treatment, while the protein concentration of ACAT2 was not affected (Extended Data Figure 3J). This suggested that ACAT1 may directly regulate 4‐Ac‐GABA production. Indeed, consistent with this, knockout of Acat1 in the mouse Hepa1‐6 cells significantly reduced 4‐Ac‐GABA (Figure 4I). At the same time, ACAT1 protein was significantly down‐regulated after PMVK knockout (Figure 4J,K). Co‐immunoprecipitation experiments with endogenous proteins showed that PMVK directly interacted with ACAT1 (Figure 4L,M) and that the C‐terminal of ACAT1 mediates this association (Extended Data Figure 3K,L). GST pull‐down assay also demonstrates the interaction of PMVK with ACAT1 (Extended Data Figure 3M). There was no interaction between GAD1 and ACAT1 (Extended Data Figure 3N,O). These results suggest that PMVK‐regulated GAD1 and ACAT1 are required for 4‐Ac‐GABA production.

### 2.4 4‐Ac‐GABA Inhibits CD8^+^ T Cell Activation and Infiltration and is Responsible for HCC Immune Escape

**Figure 5:**
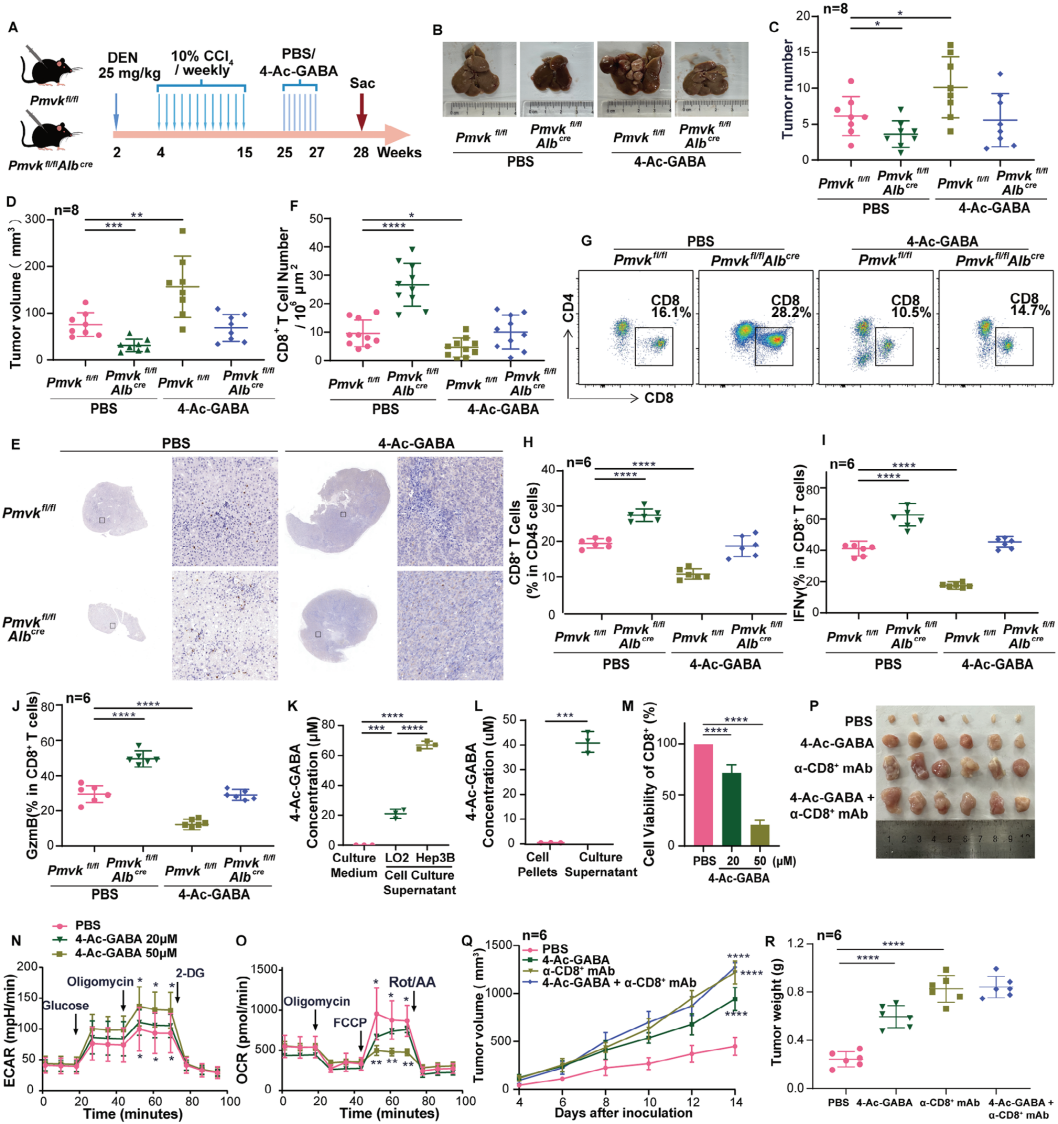
4‐Ac‐GABA inhibits CD8^+^ T cell infiltration and activation and promotes HCC immune escape. A) Schematic overview of DEN/CCl_4_ induced HCC mice model with administration of PBS or 4‐Ac‐GABA. *n = *8 mice per group. B) Representative livers from the indicated mice from (A). *n = *8 mice per group. C) Tumor number of each liver from (A). D) Average tumor volume of livers from (A). E) Representative immunohistochemical staining of CD8^+^ from (A). Scale bar, 100 µm. The immunohistochemical staining experiment was performed twice. F) Cell number in 10^6^ µm^2^ area image from (E). *n = *10 images per group. G) Pictures of flow cytometry gating of CD8^+^ T cells in DEN/CCl_4_ induced HCC mice model from (A). Cells were stained as described in Materials and Methods and doublets were excluded using FSC‐A and FSC‐W. Upper panels show percentages of CD8^+^ CD45^+^ T cells. H) Flow cytometry showed percentages of CD8^+^ CD45^+^ T cells from (A). *n = *6 mice per group. I) Percentages of IFNγ‐expressing CD8^+^ T cells from DEN/CCl_4_‐induced HCCs. *n = *6 mice per group. J) Percentages of GzmB‐expressing CD8^+^ T cells from DEN/CCl_4_‐induced HCCs. *n = *6 mice per group. K) The levels of 4‐Ac‐GABA in normal medium and Hep3B medium, as measured by UPLC‐MS/MS. *n = *3 samples per group. L) 4‐Ac‐GABA levels in CD8^+^ T cell pellets and medium, as measured by UPLC‐MS/MS. *n = *3 samples per group. M) Viability of splenic CD8^+^ T cells following treatment with 4‐Ac‐GABA as measured by MTT. N,O) ECAR and OCR analyses of splenic CD8^+^ T cells following treatment with 4‐Ac‐GABA. P) Images of Hepa1‐6 tumor xenografts from immunocompetent mice following the indicated treatments. *n = *6 mice per group. Q) Subcutaneous xenograft tumor volumes from (P). *n = *6 mice per group. R) Subcutaneous xenograft tumor weights from (P). *n = *6 mice per group.

To explore the effect of 4‐Ac‐GABA on anti‐tumor immunity, PMVK‐CKO C57BL/6J mice bearing DEN/CCl_4_‐induced HCCs were treated with 4‐Ac‐GABA, which significantly enhanced tumor growth (**Figure** **5**A–D). Immunohistochemical staining and flow cytometric evaluation also showed that the infiltration of CD8^+^ T cells was significantly reduced (Figure 5E–H). In CD8^+^ T cells isolated from tumors, PMVK inhibition led to a clear increase in the levels of IFNγ and GzmB (Figure 5I,J, Extended Data Figure 4A), whereas the levels of PD‐1, TIM‐3, and CTLA‐4 did not significantly change (Extended Data Figure 4B–F). 4‐Ac‐GABA treatment partially rescued phenotypes caused by PMVK deletion (Figure 5A–F, Extended Data Figure 4A–F). These results suggested that 4‐Ac‐GABA may affect tumor growth by reducing the activation and infiltration of CD8^+^ T cells.

What is the source of 4‐Ac‐GABA in the TME in tumors with elevated levels of PMVK? PMVK is highly expressed in Hep3B cells and a large accumulation of 4‐Ac‐GABA was found in tissue culture supernatants (Figure 5K). When splenic CD8^+^ T cells from C57BL/6J mice were treated with 4‐Ac‐GABA, there was almost no detectable 4‐Ac‐GABA in the CD8^+^ T cell pellets, and most 4‐Ac‐GABA remained in the culture medium supernatant (Figure 5L). Similar results were obtained in DEN/CCl_4_‐induced HCCs. 4‐Ac‐GABA levels were significantly reduced in HCCs arising in PMVK KO livers (Extended Data Figure 4G). Moreover, the viability of CD8^+^ T cells was decreased in a manner that was 4‐Ac‐GABA dose‐dependent (Figure 5M, Extended Data Figure 4H). CD8^+^ T cells treated with 4‐Ac‐GABA showed increased extracellular acidification rates (ECARs) and reduced oxygen consumption rates (OCRs) (Figure 5N,O). Depletion of CD8^+^ T cells in transplanted tumors in C57BL/6J immunocompetent mice resulted in faster tumor growth, which was consistent with previous results. Furthermore, 4‐Ac‐GABA treatment did not further affect CD8^+^ T cell‐depleted tumor growth (Figure 5P–R). These results suggest that the elevated PMVK levels in tumor cells produce high levels of 4‐Ac‐GABA in the TME, which suppresses the activation and infiltration of CD8^+^ T cells.

Our previous studies have demonstrated that 4‐Ac‐GABA modulates the infiltration and activity of CD8^+^ T cells within the TME. However, the precise underlying mechanism by which this is achieved remains elusive. A significant enhancement of transplanted HCC growth was observed in immunocompetent mice treated with 4‐Ac‐GABA whereas no significant effect was observed when the mice were immune‐deficient (Extended Data Figure 5A–F). This strongly suggested that 4‐Ac‐GABA influences tumor growth by impacting tumor immunity.

**Figure 6:**
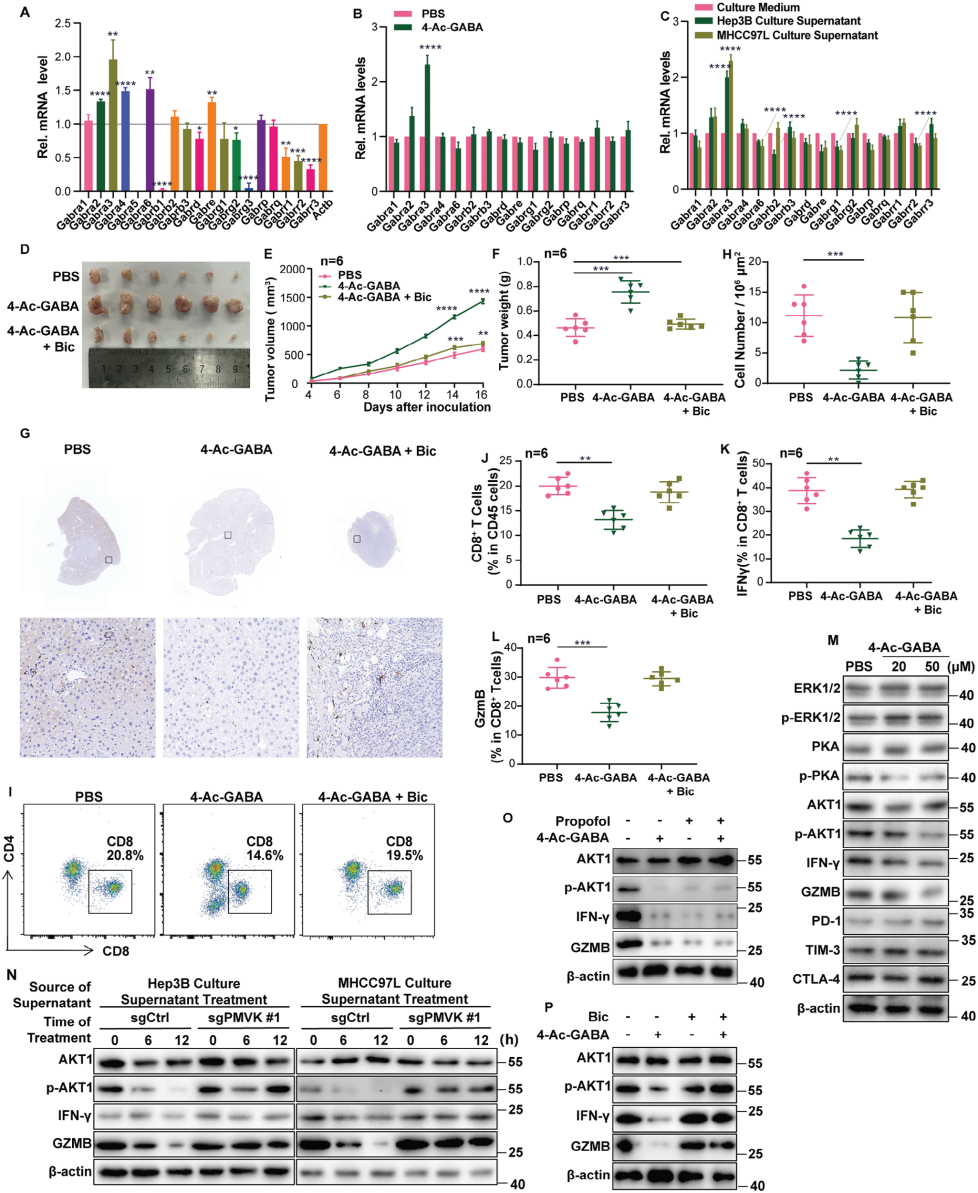
Blocking the 4‐acetaminobutyric acid receptor abolishes the effects of 4‐Ac‐GABA on CD8^+^ T cell infiltration and HCC immune escape. A) qRT‐PCR analysis of GABA receptor genes in splenic CD8^+^ T cells. ACTB transcript levels were used as a reference. *n = * 3. Data are shown as mean ± SD. B) qRT‐PCR analysis of GABA receptor genes in splenic CD8^+^ T cells from 4‐Ac‐GABA‐ treated or control mice, *n = * 3. C) qRT‐PCR analysis of GABA receptor genes in splenic CD8^+^ T cells treated with MHCC97L medium or Hep3B medium, *n = * 3. D) Subcutaneous Hepa1‐6 tumor xenografts from immunocompetent treated with the indicated agents, *n = *6 mice per group. E) Volumes of the tumors from (D). F) Tumor weights from (D). G) Representative immunohistochemical staining of CD8^+^ T cells from the Subcutaneous xenograft in (D). Scale bar, 100 µm. The immunohistochemical staining experiment was performed twice. H) Cell number in 10^6^ µm^2^ area image from (D). *n = *10 images per group. I) Flow cytometry gating of CD8^+^ T cells from subcutaneous xenograft. J) Flow cytometry showing percentages of CD8^+^ CD45^+^ T cells from (I). *n = *6 mice per group. K) Percentages of IFNγ‐expressing of CD8^+^ T cells from the xenograft shown in (I). *n = *6 mice per group. L) Percentages of GzmB of CD8^+^ T cells from the xenografts from (F). *n = *6 mice per group. M) Splenic CD8^+^ T cells from mice treated with 4‐Ac‐GABA were used for western blotting. N) Splenic CD8^+^ T cells exposed to different media were used for western blotting. O) Splenic CD8^+^ T cells treated with 4‐Ac‐GABA and propofol, a specific agonist of GABA type A receptors alpha subunit, were subjected to western blot. P) Splenic CD8^+^ T cells treated with 4‐Ac‐GABA and Bic were subjected to western blot.

We next investigated the mechanism by which 4‐Ac‐GABA affects CD8^+^ T cell activation and infiltration. Considering previous reports indicating that 4‐Ac‐GABA is an acetylation product of GABA, we asked whether the two molecules share common receptors and found high overall expression of the α subunits of GABAAR in CD8^+^ splenic T cells (**Figure** **6**A).^[^ ^19^ ^]^ The addition of 4‐Ac‐GABA to the medium resulted in a substantial increase in mRNA levels specifically for the GABAARα3 subunit (Figure 6B). The results obtained from culturing isolated CD8^+^ T cells treated with concentrated hepatoma cell medium were consistent with these findings (Figure 6C). Treatment with bicuculline (Bic), a GABAARα antagonist, effectively suppressed the accelerated tumor growth that is otherwise normally seen in response to 4‐Ac‐GABA treatment (Figure 6D–H). Flow cytometric analysis results also aligned with our earlier findings (Figure 6I–L, Extended Data Figure 5G–L). Collectively, these data suggest that 4‐Ac‐GABA impacts CD8^+^ T cells signaling pathways via surface‐bound GABAAR α3 subunits leading to reduced CD8^+^ T activation and infiltration into the TME.

### 2.5 Blocking the 4‐acetaminobutyric Acid Receptor Abolishes CD8^+^ T Cell Activation, Infiltration, and HCC Immune Escape

Previous studies have shown that stimulation of the GABAAR α3 subunit on the cell surface can affect AKT,^[^ ^9^, ^20^ ^]^ ERK,^[^ ^21^ ^]^ and PKA^[^ ^22^ ^]^ signaling pathways in cells.^[^ ^23^ ^]^ When CD8^+^ splenic T cells were treated with 4‐Ac‐GABA, the level of phosphorylated AKT1 (pAKT) was decreased, while phosphorylated ERK and PKA were unchanged (Figure 6M). Similar results were obtained in CD8^+^ T cells cultured with concentrated hepatoma cell medium (Extended Data Figure 5M). In contrast, there was no change in the pAKT1 of CD8^+^ T cells cultured with concentrated PMVK knockout hepatoma cell medium (Figure 6N). Treatment of CD8^+^ T cells with propofol, an GABAAR α subunit‐targeted activator, similarly decreased pAKT1 levels (Figure 6O) while treatment with 4‐Ac‐GABA and Bic, an GABAAR α subunit inhibitor, did not affect pAKT1 (Figure 6P). In addition, deletion of PMVK in mouse liver did not change the level of GABA in tumor tissues (Figure 3C). These results suggested that 4‐Ac‐GABA secreted by tumor cells inhibits CD8^+^ T cell activation by stimulating the GABAAR α3 subunit, thereby inhibiting the intracellular AKT1 signaling pathway.

### 2.6 PMVK is Overexpressed by Human HCCs and Correlates with Poor Clinical Outcomes

**Figure 7:**
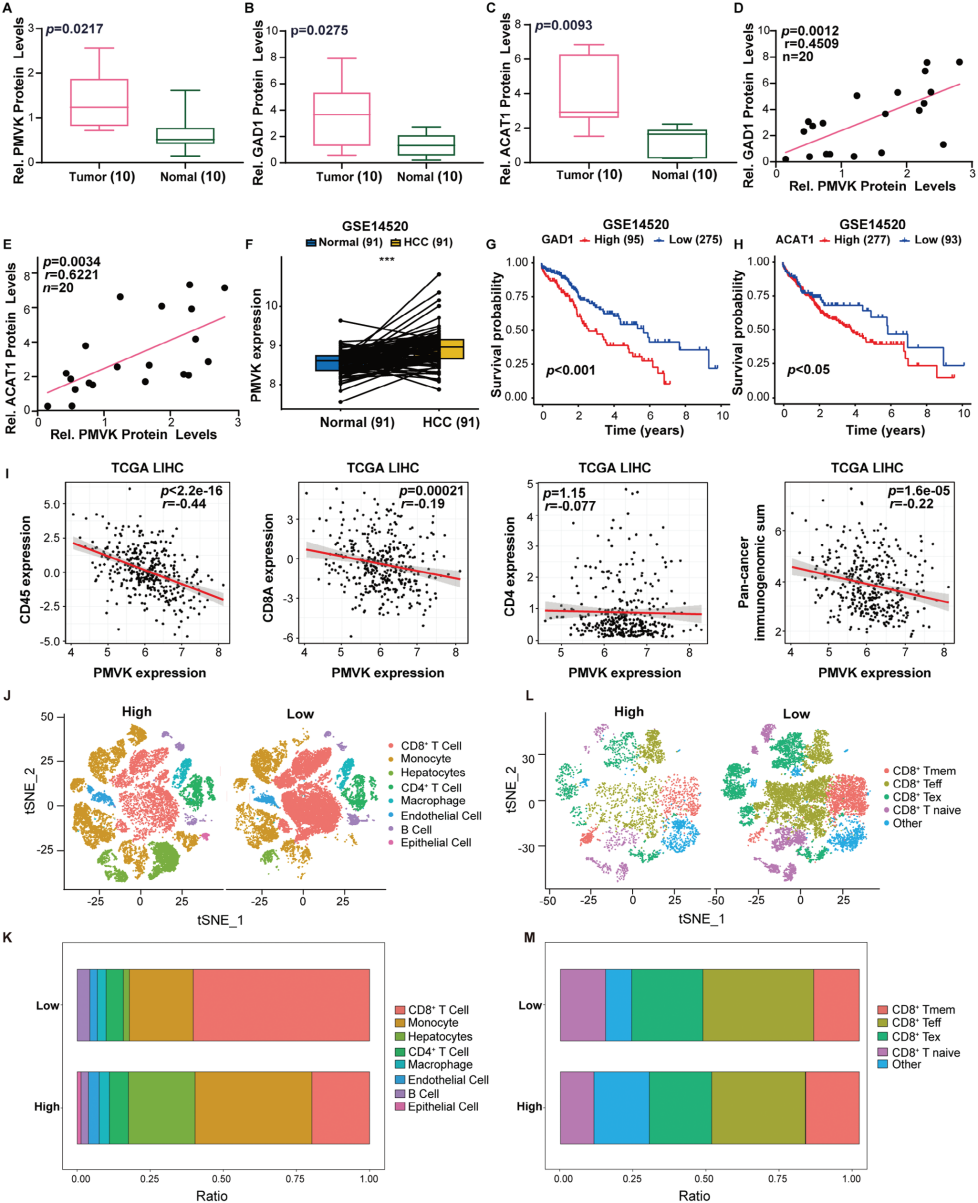
PMVK is overexpressed in human HCCs and correlates with poor clinical outcomes. A–C) Densitometric quantification of PMVK, GAD1, and ACAT1 for Extended Data Figure 6A. β‐actin was used as a normalizer. Data are shown as mean ± SD. D) Correlation of PMVK and GAD1 protein levels in HCC tissues from Extended Data Figure 6A. Each point is an individual sample. E) Correlation of PMVK and ACAT1 protein levels in HCC tissues from Extended Data Figure 6A. Each point is an individual sample. F) PMVK mRNA levels were analyzed in paired HCC tissues from GSE14520. G) Kaplan–Meier curves with univariate analysis indicating survival of patients with HCC based on high versus low expression of GAD1from GSE14520. H) Kaplan–Meier curves with univariate analysis indicating survival of patients with HCC based on high versus low expression of ACAT1 from GSE14520. I) TCGA database was used to analyze the correlation between PMVK and CD45 T cells, CD8 T cells, CD4 cells, and pan‐tumor immunogenomic sum in liver cancer tissues. J) tSNE plot from high (left, *n = *42 samples) or low (right, *n = *9 samples) expression patients. K) Bar plot of proportional differences in immune cells between the PMVK high (left, *n = *42 samples) and PMVK low (right, *n = *9 samples) groups. L) Re‐clustering of CD8^+^ T lymphocytes, tSNE visualization, and marker‐based annotation of CD8^+^ T lymphocyte subtypes, colored by cluster identity. PMVK high group, left, *n = *18 465 cells. PMVK low group, right, *n = *18 806 cells. M) Bar plot of proportional differences in CD8^+^ T lymphocytes between the PMVK high (left, *n = *3634 samples) and PMVK low (right, *n = *11 332 samples) groups. CD8^+^ Tmem, memory CD8^+^ T cells; CD8^+^ Teff, effector CD8^+^ T cells; CD8^+^ Tex, exhausted CD8^+^ T cells; CD8^+^ T naive, naive CD8^+^ T cells.

In tumor samples from 10 HCC patients, PMVK, GAD1, and ACAT1 were significantly upregulated relative to matched normal liver tissues (**Figure** **7**A–C, Extended Data Figure 6A,B). PMVK levels also positively correlated with GAD1 and ACAT1 levels (Figure 7D,E, Extended Data Figure 6C). Additionally, PMVK was also highly expressed in HCC tissues from the GEO database (Figure 7F). Finally, the upregulation of PMVK, GAD1, and ACAT1 each correlated inversely with the survival of HCC patients (Figure 7G,H, Extended Data Figure 1A). TCGA database analysis showed that PMVK expression was negatively correlated with CD8^+^ T cells in liver cancer tissues (Figure 7I).^[^ ^24^ ^]^

To further explore the relationship between *PMVK* and the TME of human HCC patients, single‐cell RNA sequencing data were analyzed from 51 HCC patients (Figure 7J). CD8^+^ T cell signatures were significantly increased in tumors with low *PMVK* expression (Figure 7J,K). Based on classical markers, CD8^+^ T cells were reclassified into four subpopulations: memory T cells (Tmem), effector T cells (Teff), exhausted T cells (Tex), and naive T cells (Figure 7L). The proportion of Teffs was dramatically up‐regulated in HCCs with low *PMVK* expression (Figure 7L,M). Together, these data suggest that PMVK is a potential immune therapeutic target for HCC.

### 2.7 Combined Targeting of PMVK and PD‐1 is Therapeutically Effective Against HCC

**Figure 8:**
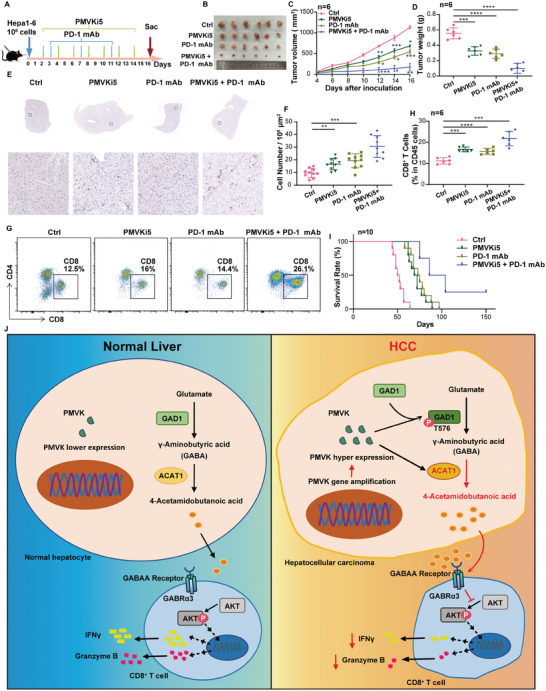
Combined treatment with a PMVK inhibitor and an anti‐PD‐1 mAb significantly increases HCC survival. A) Immunocompetent mice bearing with Hepa1‐6 xenografts. PMVK inhibitor PMVKi5 was administered every 2 days. Anti PD‐1 antibody (PD‐1 mAb) was administered every 3 days. *n = *6 mice per group. B) Subcutaneous xenograft from (A) after treatment. *n = *6 mice per group. C) Xenograft tumor volumes from (A). D) Xenograft tumor weights from (A). E) Representative immunohistochemical staining of CD8^+^ T cells from the tumors in (A). Scale bar, 100 µm. Immunohistochemical staining was performed twice. F) Cell number in 10^6^ μm^2^ area image from (E). *n = *10 images per group. G) Flow cytometry showed percentages of CD8^+^CD45^+^ T cells from (A). *n = *6 mice per group. H) Pictures of flow cytometry gating of CD8^+^ T cells in xenografts from (A). Upper panels show percentages of CD8^+^CD45^+^ T cells. *n = *6 mice per group. I) Survival curves of mice bearing xenografts and treated with the indicated combinations of PMVKi5 and anti‐PD‐1 mAb. *n = *10 mice per group. J) Schematic representation of the mechanism.

PMVK regulates 4‐Ac‐GABA levels in tumor cells while also inhibiting the infiltration and activation of TME‐associated CD8^+^ T cells. Therefore, therapy with an anti‐PD‐1 antibody combined with the PMVK inhibitor PMVKi5 was attempted in a mouse transplant model of HCC (**Figure** **8**A). Whereas treatment with either modality alone had only modest effects on tumor growth, the combination of the two was significantly more potent (Figure 8B–D). Although each treatment alone increased tumor‐infiltrating CD8^+^ T cells number, combination therapy was particularly effective in doing so (Figure 8E–H). Finally, combined treatment significantly prolonged the survival of tumor‐bearing mice (Figure 8I).

## 3 Discussion

During the course of tumorigenesis, immune cells and metabolites residing within the TME are reprogrammed and altered so as to form an immunosuppressive network, thereby efficiently inhibiting tumor‐specific immunity and promoting tumor growth.^[^ ^25^ ^]^ Because tumor and immune cells are both exposed to the TME, they will compete for key nutrients, often to the latter's disadvantage.^[^ ^6^ ^]^ Immunomodulatory molecules secreted by tumor cells into the TME can play roles beyond metabolic signaling to support immune cell development and activity, thereby altering T‐cell immunity.^[^ ^26^ ^]^ For example, large accumulations of fat have been observed in tumor cells and various immune cell subsets in the TME.^[^ ^27^ ^]^ High expression of CD36 in tumor‐infiltrating CD8^+^ T cells increases the uptake of fatty acids and oxidized low‐density lipoprotein, which leads to the accumulation of lipids in CD8^+^ T cells and their ensuing dysfunction.^[^ ^28^ ^]^ IFNγ produced by CD8^+^ T cells can inhibit tumor cells and induce SLC7A11 and ACSL4, leading to the accumulation of oxidized lipids and enhancing ferroptosis induced by immunotherapy and radiotherapy.^[^ ^27^ ^]^ Other studies have shown that inhibition of SOAT1 and PCSK9, which participate in cholesterol metabolism, can enhance the anti‐tumor activity of CD8^+^ T cells, thereby enhancing immune checkpoint blockade.^[^ ^29^ ^]^ At the same time, granzyme B produced by tumor‐infiltrating CD8^+^ T cells can degrade the extracellular matrix and promote inflammation, thereby enhancing the anti‐tumor response.^[^ ^29^ ^]^ In addition to inducing p53‐independent apoptotic pathways,^[^ ^30^ ^]^ granzyme B released by CAR T cells can activate Caspase‐3, which in turn activates GSDME and leads to pyroptosis.^[^ ^31^ ^]^ Many studies have been undertaken to investigate the metabolic crosstalk between tumor cells and immune cells at the metabolic level and to link tumor‐intrinsic metabolism with immune evasion mechanisms and immune resistance. In this study, PMVK was found to be highly expressed by HCC cells. The up‐regulated PMVK phosphorylated and activated GAD1, which increased the supply of GABA. At the same time, PMVK was shown to interact with ACAT1, stabilizing the latter, and allowing it to increase the conversion of GABA to 4‐Ac‐GABA. 4‐Ac‐GABA in turn stimulated the GABA receptor α subunit on the surface of tumor‐infiltrating CD8^+^ T cells. This inhibited AKT signaling, reduced CD8^+^ T cell activation, and allowed for unrestrained tumor growth.

The mevalonate anabolic pathway, for which acetyl‐CoA is the precursor, provides metabolites for a variety of cellular processes.^[^ ^10a^ ^]^ Mevalonate is converted into substances such as cholesterol and subsequently to bile acids, lipoproteins, steroid hormones, and other products that contribute importantly to tumor progression.^[^ ^10^, ^32^ ^]^ PMVK is an intermediate metabolic enzyme in the mevalonate pathway that catalyzes the formation of mevalonic acid‐5‐pyrophosphate from mevalonic acid‐5‐phosphate.^[^ ^10a^ ^]^ PMVK also promotes HCC development by phosphorylating β‐catenin and facilitating its nuclear translocation.^[^ ^33^ ^]^ In the current work, we have shown, that PMVK participates in a novel non‐enzymatic function, by reshaping glutamine and GABA metabolism, increasing the generation of 4‐Ac‐GABA, from GABA, stimulating CD8^+^ T cell surface GABAA receptor, inhibiting CD8^+^ T cell activation and inducing HCC immune escape.

GABA has traditionally been studied as an inhibitory neurotransmitter, which reduces neuronal excitability in the mammalian central nervous system and produces a sedative effect.^[^ ^34^ ^]^ Previous studies have identified an intrinsic pathway of GABA biosynthesis in colon cancer^[^ ^35^ ^]^ and shown that high expression of GAD1 in tumor cells leads to the accumulation of GABA.^[^ ^36^ ^]^ The secreted GABA in turn activates the GABAB receptor, inhibits GSK‐3β activity, and enhances constitutive β‐catenin signaling to promote tumor growth and immune invasion.^[^ ^16^ ^]^ In the present study, the accumulation of 4‐Ac‐GABA in the TME, activates the GABAAR on the surface of CD8^+^ T cells, thereby reducing their infiltration and activation within HCCs via the inhibition of the AKT signaling pathway.

ICB is an effective mode of immunotherapy that provides significant clinical responses in some cancers.^[^ ^37^ ^]^ Immunotherapy represents an increasingly promising approach for the treatment of a variety of solid tumors, such as melanoma and non‐small cell lung cancer.^[^ ^38^ ^]^ Because HCC progression relies on the development of immune tolerance and immune surveillance escape,^[^ ^6^ ^]^ there are good reasons to consider immunotherapy as being potentially beneficial. The most commonly used immune checkpoint blockade agents target PD‐1, PD‐L1, CTLA‐4, etc^[^ ^37^ ^]^ although their use as monotherapeutic agents is usually associated with low response rates.^[^ ^39^ ^]^ Therefore, the combination of multiple immune checkpoint inhibitors or the inclusion of other non‐cross‐resistant agents has become a new standard for HCC. In this study, PMVK is identified as a potentially high‐value target that inhibits TME resident CD8^+^ T cells. We have shown that combined therapies that simultaneously target PMVK and PD‐1 can significantly inhibit tumor growth and are significantly more efficacious than individual therapies.

In conclusion, we have shown that the mevalonate pathway enzyme PMVK reprograms glutamine metabolism so as to highly favor the synthesis of 4‐Ac‐GABA. This is achieved by recruiting and activating GAD1 and ACAT1, leading to GABA accumulation, increased efficiency of 4‐Ac‐GABA synthesis, and the suppression of CD8^+^ T cell activation, intratumoral infiltration, and anti‐tumor responses. A new mechanism has been revealed whereby cancer metabolic reprogramming‐mediated alteration of metabolites in the TME significantly impacts tumor immunity (Figure 8J). PMVK inhibition, perhaps with agents such as PMVKi5 or its analogs and in combination with ICB thus represents a rational and novel therapeutic approach.

## 4 Experimental Section

### Vectors, Reagents, Antibodies, Cell Culture and Stable Cell Lines

Human PMVK sgRNAs (Table S3, Supporting Information) were expressed in the lenticrispr V2 vector as were mouse *Pmvk*, *Gad1*, and *Acat1* sgRNAs (Table S3, Supporting Information). The open reading frames of human PMVK, ACAT1, and GAD1 were amplified and cloned into pHAGE‐CMV‐MCS‐PGK‐3×FLAG and pCMV‐HA vectors. Mutations in the GAD1 cDNA sequence were generated by overlap extension PCR. Primers for constructs used in this study were listed in Table S4, Supporting Information, and were synthesized by GeneCreate Biological Engineering Co. Ltd (Wuhan, China). Transfection and the establishment of stable cell lines were performed as previously described.^[^ ^40^ ^]^ Anti‐HA magnetic beads and anti‐FLAG affinity magnetic beads were purchased from Selleck (Houston, USA) (Table S5, Supporting Information). Antibodies used in this study were listed in Table S6, Supporting Information.

Human HEK293 cells were obtained from the American Type Culture Collection in 2009. Human liver tumor‐derived cell lines MHCC97L and Hep3B were obtained from the China Center for Type Culture Collection and the Cell Bank of the Type Culture Collection of The Chinese Academy of Sciences and cultured as previously described.^[^ ^41^ ^]^ Mouse Hepa1‐6 cells were kindly provided by Prof. Yong Liu (Wuhan University). All cells were regularly authenticated by short tandem repeat analysis, were tested for the absence of Mycoplasma, and were used within 5 passages after thawing.

### Animal Experiments

All animal studies were approved by the Animal Care Committee of Wuhan University (No.WQ20210130). Four‐week‐old male BALB/c nude mice were purchased from Gempharmatech (Jiangsu, China). Pmvk^fl/fl^ mice (Cyagen Biosciences) have loxP sites flanking exons 2 and 5 of the *Pmvk* gene. Alb‐Cre mice were kindly provided by Prof. Yong Liu (Wuhan University). To generate *Pmvk* ^fl/fl^/Alb‐cre mice, *Pmvk* ^fl/fl^ mice were crossed with Alb‐Cre mice. All mouse genotype identification primer sequences used were shown in Table S7, Supporting Information. Tumor xenograft growth was monitored every three days for 16 days. Tumor volumes were calculated by the equation V (mm^3^) = *a* × *b* × *(a* + *b*)/2, where a was the length and b was the width. For induction of HCCs, C57BL/6J, *Pmvk* ^fl/fl^, and *Pmvk* ^fl/fl^/Alb‐cre male mice were intraperitoneally administrated with N‐Nitrosodiethylamine (DEN) (25 mg kg^−1^) (Sigma) at 2 weeks after birth. From 4–15 weeks, mice were intraperitoneally injected with 10% CCl_4_ (5 ml kg^‐1^) weekly. After 28 weeks, livers were examined. Littermate mice were randomly divided into control and various treatment groups. All cohorts were maintained as blinded cohorts following these treatments.

### Flow Cytometry

Cells were stained with FITC‐, PE‐, BV421‐, BV605‐, BV650‐, PerCP‐ and APC‐labeled mAbs according to the manufacturer's instructions, and flow cytometry was performed using a FACSCalibur flow cytometer (Becton‐Dickinson, Franklin Lakes, NJ). FACS data were analyzed with FlowJo software (Tree Star, Ashland, OR). The following mAbs were purchased from BD Pharmingen (San Diego, CA): PE‐CF594e CD279, PerCP‐Cy5.5 IFN‐γ, APC‐Cy7 CD45, BV421 CD3e, BV605 CD8a, BV650 CD36. PE/Cyanine7 CD152, APC CD4, and PE GRANZYME B were obtained from BioLegend (San Diego, CA). Dead cells were excluded by staining with Fixable Viability Stain 510 (BD Pharmingen). Antibodies used in this study were listed in Table S6, Supporting Information. Flow Cytometry analyses were performed as described previously.^[^ ^42^ ^]^

### Sources of Human HCC samples

Human HCC samples were used as previously described.^[^ ^35^ ^]^ All procedures that involved human HCC sample collection were approved by the ethics committee of Wuhan University (No.2022030) and the Union Hospital in Wuhan, China, and conformed to the ethical guidelines of the 1975 Declaration of Helsinki. Informed written consent of all participants was signed by all individuals or their families and obtained at the Union Hospital in Wuhan, China. The diagnoses of all samples were confirmed by histological review.

### Immunohistochemistry

Liver sections were fixed in 10% formalin and embedded in paraffin according to standard protocols. Liver sections were sectioned and stained with CD8^+^, CD4^+^, CD11c, and F4/80 markers for indicated immune cells. Images were obtained on a Leica Aperio VERSA 8 microscope.

### Co‐IP and Immunoblotting

Transfected cells were lysed in 1 mL lysis buffer [50 mM Tris (pH 7.4), 150 mm NaCl, 1% NP‐40, 0.5% Sodium deoxycholate, 0.1% SDS, 5 mM EDTA, 10 mg mL^−1^ aprotinin, 10 mg mL^−1^ leupeptin, and 1 mM PMSF]. Sepharose beads were washed three times with 1 mL lysis buffer containing 0.1% NP‐40. Co‐IP and immunoblot analysis were performed as described.^[^ ^43^ ^]^

### qRT‐PCR

Total RNA was isolated using Trizol followed by DNase treatment. Reverse transcription was performed with a cDNA Synthesis Kit and qPCR were performed using SYBR Green master mix using standard protocols. All qPCR primer sequences used are shown in Table S8, Supporting Information. β‐actin was used as an internal control.

### Protein half‐life Assay

After achieving ≈70% confluence, cells in 12‐well plates were transfected with the indicated plasmids using Lipofectamine 2000. 36 h later, the cells were treated with cycloheximide (CHX, 50 µg mL^−1^) for the indicated times before collection for immunoblot analysis.

### MTT Assay

For MTT assays, cells were seeded into 96‐well plates (10^3^ cells each well) and cultured in DMEM supplemented with 10% FBS. After 3–7 days of incubation, MTT was added and cells were incubated for 4 h at 37 °C. At the indicated time, this was replaced with DMSO (200 µL). Absorbance was then read at 570 nm using an ELX800 absorbance microplate reader.

### GST Pull‐down Assay

The Rosetta E. coli was transformed with the indicated plasmid and cultured in 2×YT medium. The cells were then induced with IPTG (1:1000) for 3 h. Lysates were incubated with Ni‐Charged magnetic beads (GenScript, L00295) or Glutathione magnetic beads (GenScript, L00327) and purified according to the manufacturer's instructions. The ACAT1 and GAD1 or its mutant fusion proteins were incubated with GST or GST‐PMVK fusion protein bound to Sepharose beads in 1 mL RIPA buffer at 4 °C for 4 h. The beads were then washed, eluted, and analyzed by IB.

### In Vitro Phosphorylation Assay

Recombinant GST‐GAD1‐His and PMVK‐His were prepared and purified from Rossita. 1 µg GST‐GAD1‐His protein was incubated with 1 µg PMVK‐His in a 50 µL kinase reaction buffer (20 mM Tris‐HCl pH 7.5, 10 mM MgCl_2_, 50 mm KCl, 30 µm ATP) at 37 °C for 0.5 h. Reactions were stopped by the addition of SDS sample buffer, and samples were then heated for 5 min at 95 °C. Phosphorylation of GAD1 was identified by immuno‐blot with GAD1 antibody.

### Extracellular Acidification Rate (ECAR) and Oxygen Consumption Rate (OCR) Assays

ECAR and OCR were measured using the Seahorse XFe24 Extracellular Flux Analyzer (Seahorse Bioscience) according to the manufacturer's instructions.^[^ ^43^ ^]^ Briefly, 3 × 10^4^ primary T cells per well were seeded into a Seahorse XF 24 cell culture microplate overnight. Following incubation in the assay medium (XF‐Base medium supplemented plus 2 mM L‐glutamine) for 1 h at 37 ^°^C (non‐CO_2_ incubator), ECAR was measured at the basal level and in response to sequential injection of glucose (10 mM), oligomycin (1 mM) and 2‐DG (50 mM) (XF Glycolysis Stress Test Kit, Agilent Technologies), respectively. OCR was measured following incubation in the assay medium (XF Base Medium supplemented with 10 mm D‐glucose, 1 mm sodium pyruvate, and 2 mm L‐glutamine) for 1 h at 37 ^°^C (non‐CO_2_ incubator) at the basal level and in response to injection of oligomycin (1 mm), FCCP (2 mm), and antimycin/rotenone (0.5 mm) (XF Cell Mito Stress Test Kit, Agilent Technologies), respectively. Data were analyzed by Seahorse XF24 Wave software. OCR was reported in pmols/minute and ECAR in mpH units/minute and normalized to cell number.

### Hydrophilic Metabolomics Analysis

After frozen samples were thawed at 4 °C, an appropriate amount was added to the pre‐cooled methanol/acetonitrile/water solution (2:2: 1, v/v), vortexed, sonicated at low temperature for 30 min, chilled at −20 °C for 10 min and centrifuged at 14 000 g at 4 °C for 20 min. The supernatant was dried under a vacuum and re‐dissolved in 100 µL aqueous acetonitrile solution (acetonitrile: water = 1:1, v/v). The sample was then vortexed and centrifuged at 14 000 g at 4 °C for 15 min and the supernatant was removed and separated on an Agilent 1290 Infinity LC ultra‐performance liquid chromatography system (UHPLC) HILIC column (Santa Clara, CA). The samples were placed in an autosampler at 4 °C throughout the analysis. In order to avoid the influence caused by the fluctuation of the instrument detection signal, the samples were consecutively analyzed in random order. Following UHPLC, samples were analyzed by mass spectrometry on a Triple TOF 6600 mass spectrometer (AB SCIEX, Framingham, MA). The original data were converted into mzXML format by ProteoWizard, and then peak alignment, retention time correction, and peak area extraction were performed by XCMS software. The data extracted by XCMS were first subjected to metabolite structure identification, data preprocessing, and then experimental data quality evaluation and final data analysis.

### Targeted Metabolic Assay of 4‐Ac‐GABA

Cell precipitates or cell culture medium were thawed on ice. 50 µL and 300 µL respectively, of precipitant (including internal standard) was added and vortexed for 10 minutes. After centrifugation at 18 000 g for 20 min at 4 °C, 80 uL of the supernatant was removed and transferred to a 96‐well plate. Finally, the plates were sealed and used for LC‐MS analysis. Reagent blanks and mixed quality control samples were used to monitor possible contamination and data quality during analysis before and after sample analysis. Raw data files generated by UPLC‐MS/MS were processed using MassLynx software (v4.1, Waters, Milford, MA, USA).

### Data Availability and Single‐cell RNA‐seq Analysis

HCC datasets were downloaded from The Cancer Genome Atlas (TCGA) data portal (http://www.tcga‐data.nci.nih.gov). PMVK and GAD1 mRNA levels were analyzed from TCGA and NCBI GEO databases (https://www.ncbi.nlm.nih.gov).

Single‐cell RNA‐seq data were obtained from the public dataset (GSE151530) in Gene Expression Omnibus (GEO) and analyzed using the Seurat package in R.^[^ ^44^ ^]^ Multiple single‐cell sample integration and batch effect correction were performed using the harmony algorithm. Single‐cell characterization scores were obtained using genomic variation analysis (GSVA) and the GSVA software package from Bioconductor. The differential gene expression and signaling pathways between the *PMVK*‐high and *PMVK*‐low groups were calculated using the limma package.

### Statistics and Reproducibility

Data were presented as mean ± SD or mean ± SEM. Log‐rank tests were used for survival analyses. Student's t‐test was used for comparisons between the two groups. One‐way ANOVA was used for multiple comparisons in patient populations. The correlation coefficient (r) and P‐values were obtained from Pearson correlation analysis. Pearson's r was calculated by GraphPad Prism 8. GraphPad Prism 8 was used for statistical calculations. *p <* 0.05 was considered to be statistically significant; *p < 0.05, **p < 0.01, ***p < 0.001, ****p < 0.0001; n.s., not significant. All experiments were repeated with a minimum of three independent repeats unless otherwise noted. Each experiment was repeated independently with similar results.

## Conflict of Interest

The authors declare that they have no conflict of interest.

## Author Contributions

X.Z. and Y.L. designed the study; X.Z. performed most of the experiments; X.Z., Y.Y., Y.C. constructed plasmids; M.Li. performed to analyze sequencing and clinical data; X.Z., Z.C. performed animal experiments. All authors discussed the results. X.Z., E.P., and Y.L. wrote the manuscript with comments from all authors.

## Supporting information



Supporting Information

Supporting Information

Supporting Information

Supporting Information

Supporting Information

## Data Availability

The data that support the findings of this study are available on request from the corresponding author. The data are not publicly available due to privacy or ethical restrictions.

## References

[1] a) A. Villanueva , New Eng. J. Med. 2019, 380, 1450; 30970190 10.1056/NEJMra1713263

[2] a) J. Bruix , M. Reig , M. Sherman , Gastroenterology 2016, 150, 835; 26795574 10.1053/j.gastro.2015.12.041

[3] a) N. D. Parikh , A. Pillai , Proc. World Congr. Gastroenterol. 2021, 19, 2020;

[4] a) G. Oliveira , C. J. Wu , Nat Rev Cancer 2023, 23, 295; 37046001 10.1038/s41568-023-00560-y PMC10773171

[5] a) A. B. El‐Khoueiry , B. Sangro , T. Yau , T. S. Crocenzi , M. Kudo , C. Hsu , T. Y. Kim , S. P. Choo , J. Trojan , T. H. R. Welling , T. Meyer , Y. K. Kang , W. Yeo , A. Chopra , J. Anderson , C. Dela Cruz , L. Lang , J. Neely , H. Tang , H. B. Dastani , I. Melero , Lancet 2017, 389, 2492; 28434648 10.1016/S0140-6736(17)31046-2 PMC7539326

[6] a) R. Donne , A. Lujambio , Hepatology 2023, 77, 1773; 35989535 10.1002/hep.32740 PMC9941399

[7] N. N. Pavlova , J. Zhu , C. B. Thompson , Cell Metab. 2022, 34, 355. 35123658 10.1016/j.cmet.2022.01.007 PMC8891094

[8] L. Satriano , M. Lewinska , P. M. Rodrigues , J. M. Banales , J. B. Andersen , Nat Rev Dis Primers 2019, 16, 748. 10.1038/s41575-019-0217-8 31666728

[9] J. Cheng , J. Yan , Y. Liu , J. Shi , H. Wang , H. Zhou , Y. Zhou , T. Zhang , L. Zhao , X. Meng , H. Gong , X. Zhang , H. Zhu , P. Jiang , Cell Metab. 2023, 35, 961. 37178684 10.1016/j.cmet.2023.04.017

[10] a) G. Gruenbacher , M. Thurnher , Cancer Lett 2015, 356 , 192; 24467965 10.1016/j.canlet.2014.01.013

[11] S. H. Moon , C. H. Huang , S. L. Houlihan , K. Regunath , W. A. Freed‐Pastor , J. P. Morris , D. F. Tschaharganeh , E. R. Kastenhuber , A. M. Barsotti , R. Culp‐Hill , W. Xue , Y. J. Ho , T. Baslan , X. Li , A. Mayle , E. de Stanchina , L. Zender , D. R. Tong , A. D'Alessandro , S. W. Lowe , C. Prives , Cell 2019, 176, 564. 30580964 10.1016/j.cell.2018.11.011 PMC6483089

[12] a) A. A. Cluntun , M. J. Lukey , R. A. Cerione , J. W. Locasale , Trends Cancer 2017, 3, 169; 28393116 10.1016/j.trecan.2017.01.005 PMC5383348

[13] a) N. J. Tillakaratne , L. Medina‐Kauwe , K. M. Gibson , Comp. Biochem. Physiol. A. 1995, 112, 247; 10.1016/0300-9629(95)00099-2 7584821

[14] H. Bao , Z. Peng , X. Cheng , C. Jian , X. Li , Y. Shi , W. Zhu , Y. Hu , M. Jiang , J. Song , F. Fang , J. Chen , X. Shu , J. Exp. Clin. Cancer Res. 2023, 42, 344. 38105184 10.1186/s13046-023-02921-9 PMC10726571

[15] M. Mazurkiewicz , A. Opolski , J. Wietrzyk , C. Radzikowski , Z. Kleinrok , J. Exp. Clin. Cancer Res. 1999, 18, 247. 10464715

[16] D. Huang , Y. Wang , J. W. Thompson , T. Yin , P. B. Alexander , D. Qin , P. Mudgal , H. Wu , Y. Liang , L. Tan , C. Pan , L. Yuan , Y. Wan , Q. J. Li , X. F. Wang , Nat. Cell Biol. 2022, 24, 230. 35145222 10.1038/s41556-021-00820-9 PMC8852304

[17] N. G. Bowery , G. P. Jones , Br. J. Pharmacol. 1976, 56, 323. 1260178 10.1111/j.1476-5381.1976.tb07646.x PMC1666980

[18] a) K. Cui , S. Yao , H. Zhang , M. Zhou , B. Liu , Y. Cao , B. Fei , S. Huang , Z. Huang , Oncogene 2021, 40, 2130; 33627780 10.1038/s41388-021-01677-w

[19] R. Moreau , J. Clària , F. Aguilar , F. Fenaille , J. J. Lozano , C. Junot , B. Colsch , P. Caraceni , J. Trebicka , M. Pavesi , C. Alessandria , F. Nevens , F. Saliba , T. M. Welzel , A. Albillos , T. Gustot , J. Fernández , C. Moreno , M. Baldassarre , G. Zaccherini , S. Piano , S. Montagnese , V. Vargas , J. Genescà , E. Solà , W. Bernal , N. Butin , T. Hautbergue , S. Cholet , F. Castelli , et al., J Hepatol 2020, 72, 688. 31778751 10.1016/j.jhep.2019.11.009

[20] K. Gumireddy , A. Li , A. V. Kossenkov , M. Sakurai , J. Yan , Y. Li , H. Xu , J. Wang , P. J. Zhang , L. Zhang , L. C. Showe , K. Nishikura , Q. Huang , Nat. Commun. 2016, 7, 1071. 10.1038/ncomms10715 PMC4754346 26869349

[21] N. Tyagi , W. Gillespie , J. C. Vacek , U. Sen , S. C. Tyagi , D. Lominadze , J. Cell. Physiol. 2009, 220, 257. 19308943 10.1002/jcp.21757 PMC2811271

[22] I. Kaushik , S. K. Srivastava , Mol. Therapy: J. Am. Soc. Gene Therapy 2022, 30, 2584. 10.1016/j.ymthe.2022.03.012 PMC9263240 35331907

[23] Y. Yang , L. Ren , W. Li , Y. Zhang , S. Zhang , B. Ge , H. Yang , G. Du , B. Tang , H. Wang , J. Wang , Biomed. Pharmacother. 2023, 161, 114410. 36812710 10.1016/j.biopha.2023.114410

[24] L. Ma , M. O. Hernandez , Y. Zhao , M. Mehta , B. Tran , M. Kelly , Z. Rae , J. M. Hernandez , J. L. Davis , S. P. Martin , D. E. Kleiner , S. M. Hewitt , K. Ylaya , B. J. Wood , T. F. Greten , X. W. Wang , Cancer Cell 2019, 36, 418. 31588021 10.1016/j.ccell.2019.08.007 PMC6801104

[25] I. Martínez‐Reyes , N. S. Chandel , Nat Rev Cancer 2021, 21, 669. 34272515 10.1038/s41568-021-00378-6

[26] W. Zou , D. R. Green , Cell Metab. 2023, 35, 1101. 37390822 10.1016/j.cmet.2023.06.003 PMC10527949

[27] C. Fang , T. Weng , S. Hu , Z. Yuan , H. Xiong , B. Huang , Y. Cai , L. Li , X. Fu , Oncoimmunology 2021, 10, 1962591. 34408924 10.1080/2162402X.2021.1962591 PMC8366549

[28] A. J. Oliver , A. S. Davey , S. P. Keam , S. Mardiana , J. D. Chan , B. von Scheidt , P. A. Beavis , I. G. House , J. R. Van Audernaerde , P. K. Darcy , M. H. Kershaw , C. Y. Slaney , Clin. Transl. Immunol. 2019, 8, e1094. 10.1002/cti2.1094 PMC6869967 31768254

[29] X. Ma , E. Bi , Y. Lu , P. Su , C. Huang , L. Liu , Q. Wang , M. Yang , M. F. Kalady , J. Qian , A. Zhang , A. A. Gupte , D. J. Hamilton , C. Zheng , Q. Yi , Cell Metab. 2019, 30, 143. 31031094 10.1016/j.cmet.2019.04.002 PMC7061417

[30] T. Ben Safta , L. Ziani , L. Favre , L. Lamendour , G. Gros , F. Mami‐Chouaib , D. Martinvalet , S. Chouaib , J. Thiery , J. Immunol. 2015, 194, 418. 25404359 10.4049/jimmunol.1401978

[31] Y. Liu , Y. Fang , X. Chen , Z. Wang , X. Liang , T. Zhang , M. Liu , N. Zhou , J. Lv , K. Tang , J. Xie , Y. Gao , F. Cheng , Y. Zhou , Z. Zhang , Y. Hu , X. Zhang , Q. Gao , Y. Zhang , B. Huang , Sci Immunol 2020, 5, aax7969. 10.1126/sciimmunol.aax7969 31953257

[32] P. J. Mullen , R. Yu , J. Longo , M. C. Archer , L. Z. Penn , Nat Rev Cancer 2016, 16, 718. 27562463 10.1038/nrc.2016.76

[33] Z. Chen , X. Zhou , X. Zhou , Y. Tang , M. Lu , J. Zhao , C. Tian , M. Wu , Y. Liu , E. V. Prochownik , F. Wang , Y. Li , Advanced science 2023, 10, e2204909. 36808719 10.1002/advs.202204909 PMC10131864

[34] W. Koh , H. Kwak , E. Cheong , C. J. Lee , Nat Rev Neurosci 2023, 24, 523. 37495761 10.1038/s41583-023-00724-7

[35] D. Huang , P. B. Alexander , Q. J. Li , X. F. Wang , Trends Cell Biol. 2023, 33, 403. 36114091 10.1016/j.tcb.2022.08.004 PMC10008753

[36] Z. Kleinrok , M. Matuszek , J. Jesipowicz , B. Matuszek , A. Opolski , C. Radzikowski , J Physiol. Pharmacol 1998, 49, 303. 9670113

[37] Y. Diesendruck , I. Benhar , Drug Resist. Updat. 2017, 30, 39. 28363334 10.1016/j.drup.2017.02.001

[38] N. Xie , G. Shen , W. Gao , Z. Huang , C. Huang , L. Fu , Signal Transduct. Targeted Therapy 2023, 8, 9. 10.1038/s41392-022-01270-x PMC9816309 36604431

[39] T. E. Keenan , S. M. Tolaney , J Natl Compr Canc Netw 2020, 18, 479. 32259782 10.6004/jnccn.2020.7554

[40] L. Gu , Y. Zhu , X. Lin , B. Lu , X. Zhou , F. Zhou , Q. Zhao , E. V. Prochownik , Y. Li , Hepatology 2021, 73, 160. 32221968 10.1002/hep.31249

[41] L. Gu , Y. Zhu , X. Lin , Y. Li , K. Cui , E. V. Prochownik , Y. Li , Cancer Res. 2018, 78, 5808. 30143522 10.1158/0008-5472.CAN-18-0340

[42] H. Radziewicz , C. C. Ibegbu , H. Hon , N. Bédard , J. Bruneau , K. A. Workowski , S. J. Knechtle , A. D. Kirk , C. P. Larsen , N. H. Shoukry , A. Grakoui , J. Immunol. 2010, 184, 2410. 20100932 10.4049/jimmunol.0902994 PMC2924663

[43] Y. Zhu , L. Gu , X. Lin , C. Liu , B. Lu , K. Cui , F. Zhou , Q. Zhao , E. V. Prochownik , C. Fan , Y. Li , Mol. Cell 2020, 77, 138. 31735643 10.1016/j.molcel.2019.10.015

[44] X. Zhou , G. Wang , C. Tian , L. Du , E. V. Prochownik , Y. Li , Nat. Commun. 2024, 15, 5851. 38992029 10.1038/s41467-024-50138-x PMC11239938

