# Supplementary material for: Increases in 4‐Acetaminobutyric Acid Generated by Phosphomevalonate Kinase Suppress CD8+ T Cell Activation and Allow Tumor Immune Escape
Source: Adv Sci (Weinh). 2024 Sep 26;11(43):2403629. doi: 10.1002/advs.202403629 (PMC11578309; doi:10.1002/advs.202403629)
Supplement: Supplementary file 1 — Supporting Information [file ADVS-11-2403629-s002.pdf]

ADVANCED SCIENCE
Open Access

Supporting Information

for *Adv. Sci.*, DOI 10.1002/advs.202403629

Increases in 4-Acetaminobutyric Acid Generated by Phosphomevalonate Kinase Suppress $CD8^{+}$ T Cell Activation and Allow Tumor Immune Escape

*Xinyi Zhou, Zhiqiang Chen, Yijiang Yu, Mengjiao Li, Yu Cao, Edward V. Prochownik and Youjun Li**

Extended Data

Increases in 4-Acetaminobutyric Acid Generated by Phosphomevalonate Kinase Suppress $CD8^+$ T Cell Activation and Allow Tumor Immune Escape

Extended Data Figure 1

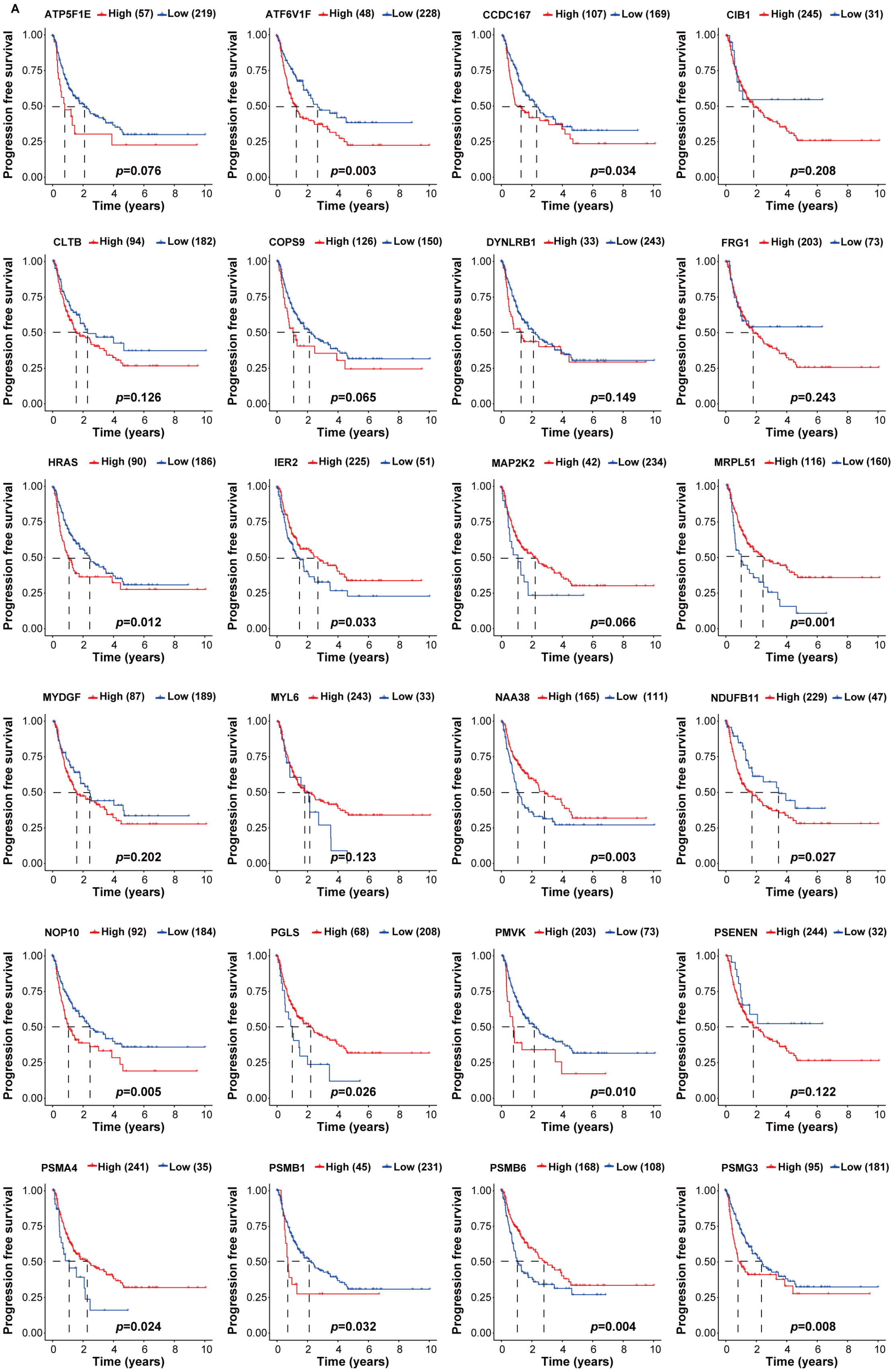

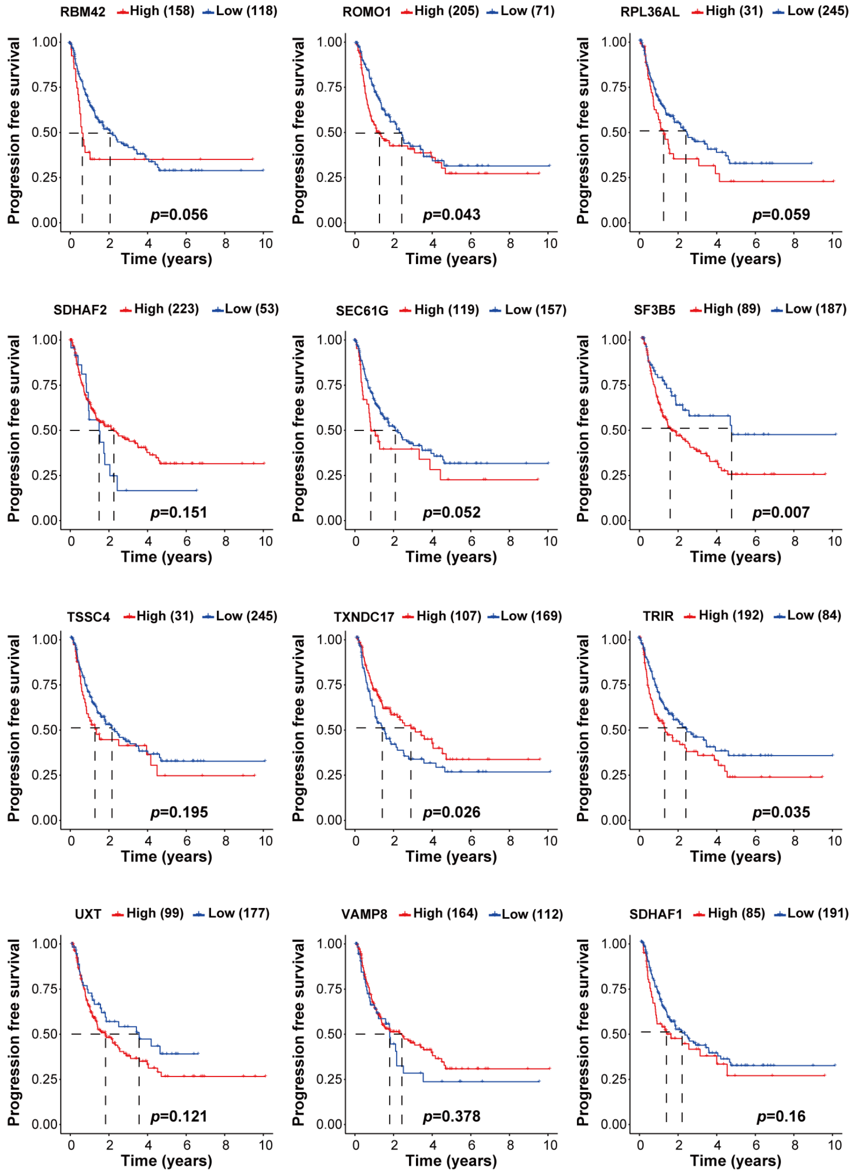

B

Gene	P-Value
MRPL51	0.001
ATP6V1F	0.003
NAA38	0.003
PSMB6	0.004
NOP10	0.005
SF3B5	0.007
PSMG3	0.008
PMVK	0.010
HRAS	0.012
PSMA4	0.024
PGLS	0.026
TXNDC17	0.026
NDUFB11	0.027
PSMB1	0.032
IER2	0.033
CCDC167	0.034
TRIR	0.035
ROMO1	0.043
SEC61G	0.052
RBM42	0.056
RPL36AL	0.059
COPS9	0.065
MAP2K2	0.066
ATP5F1E	0.076
UXT	0.121
PSENEN	0.122
MYL6	0.123
CLTB	0.126
DYNLRB1	0.149
SDHAF2	0.151
SDHAF1	0.167
TSSC4	0.195
MYDGF	0.202
CIB1	0.208
FRG1	0.243
VAMP8	0.378

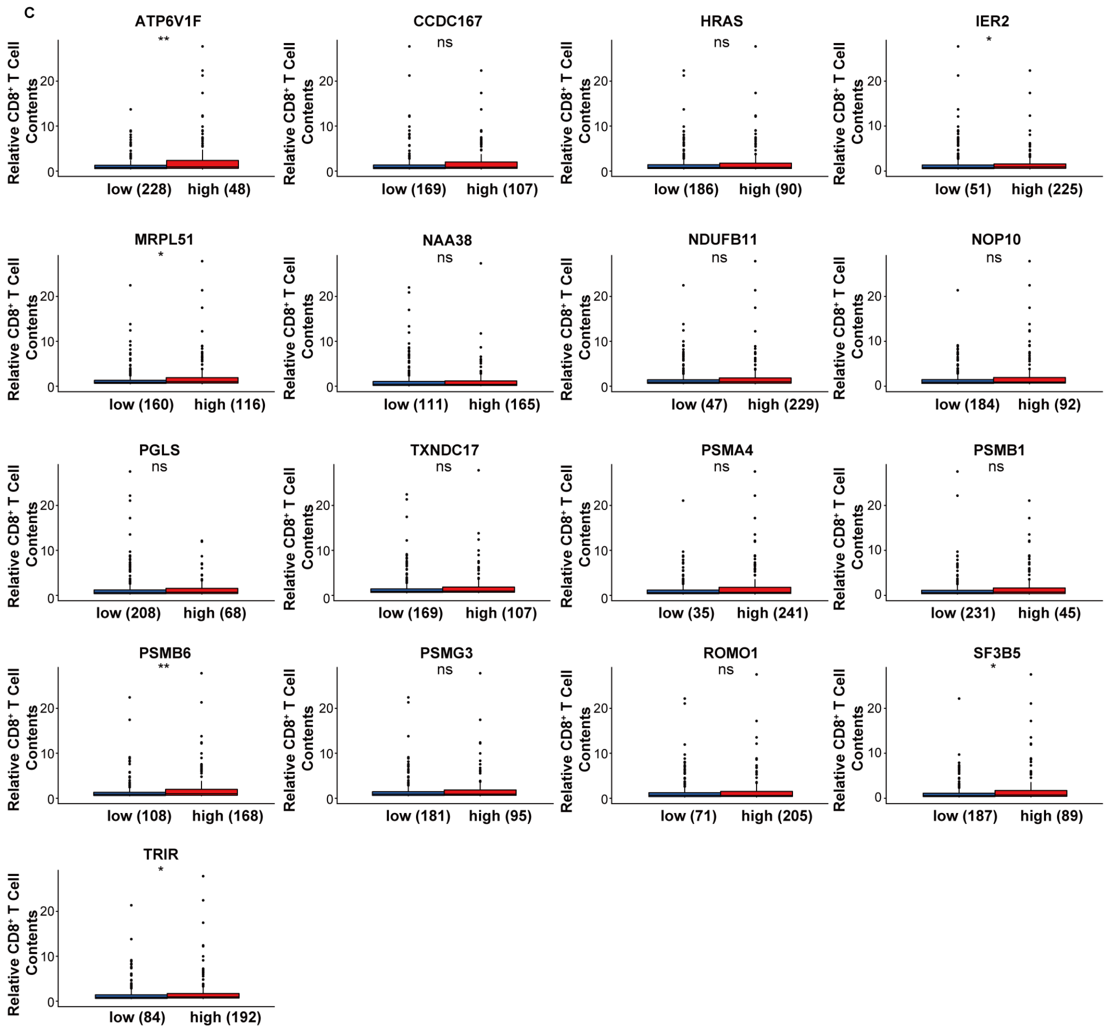

Extended Data Figure 1. PMVK expression correlates with HCC immune escape by WGCNA.

(**A**) Progression free survival analysis. (**B**) P-value statistics of progression free survival in (**A**). (**C**) $CD8^+$ T cell content analysis was performed on the expression matrix of genes in HCC tissues. *$p < 0.05$, **$p < 0.01$, ns, not significant.

Extended Data Figure 2

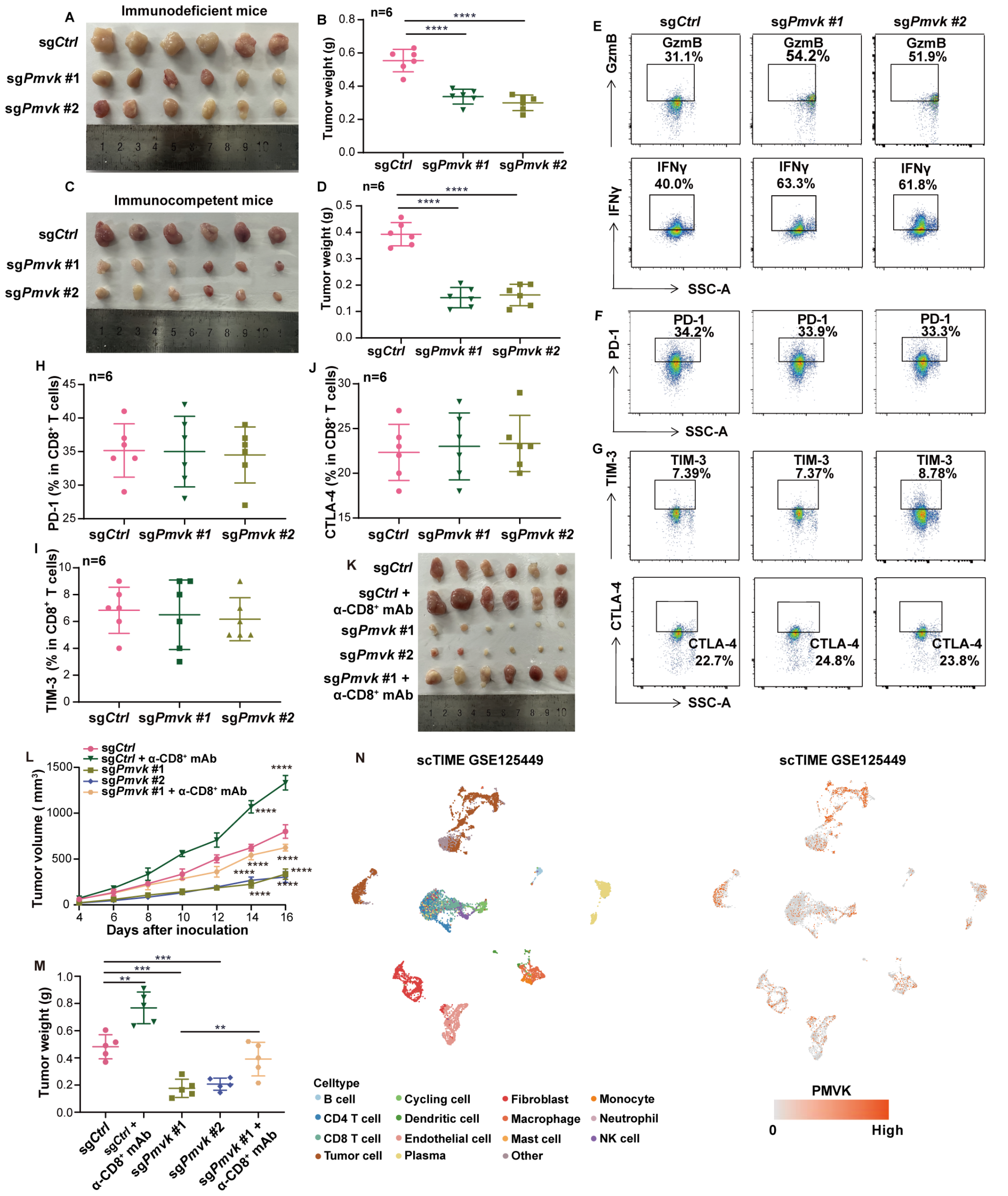

Extended Data Figure 2. PMVK deficiency enhances CD8$^+$ T cell infiltration and activation and inhibits tumor immune escape.

(**A**) Images of Hepa1-6 xenograft experiments performed in immunodeficient mice. Hepa1-6 cells expressed the indicated plasmids from Figure 1**A**. n = 6 mice per group.

(**B**) Tumor weights from Figure **2G**. (**C**) Individual Hepa1-6 subcutaneous xenografts from immunocompetent mice. Hepa1-6 cells expressed the indicated plasmids from Figure **2I**. n = 6 mice per group. (**D**) Tumor weights from Figure **2I**. (**E-G**) Flow cytometry showing percentages of GZMB-, IFNγ- (**E**), PD-1- (**F**), TIM-3- and CTLA-4- (**G**) expressing $CD8^+$ T cells in xenograft from (**C**). n = 6 mice per group. (**H-J**) percentages of PD-1- (**H**), TIM-3- (**I**) and CTLA-4- (**J**) expressing $CD8^+$ T cells in xenograft from (**C**). n = 6 mice per group. Data are shown as mean ± SD. (**K**) Images of Hepa1-6 xenografts from immunocompetent mice in treated with anti-$CD8^+$ T cell antibody. n = 6 mice per group. (**L**) Xenograft tumor volumes from (**K**). n = 6 mice per group. (**M**) Xenograft tumor weights from (**K**). n = 6 mice per group. (**N**) The level of PMVK in different types of cells in liver cancer tissues was analyzed using the single cell dataset (GSE125449) of the GSE database. $^{**}p < 0.01$, $^{***}p < 0.001$, $^{****}p < 0.0001$.

Extended Data Figure 3

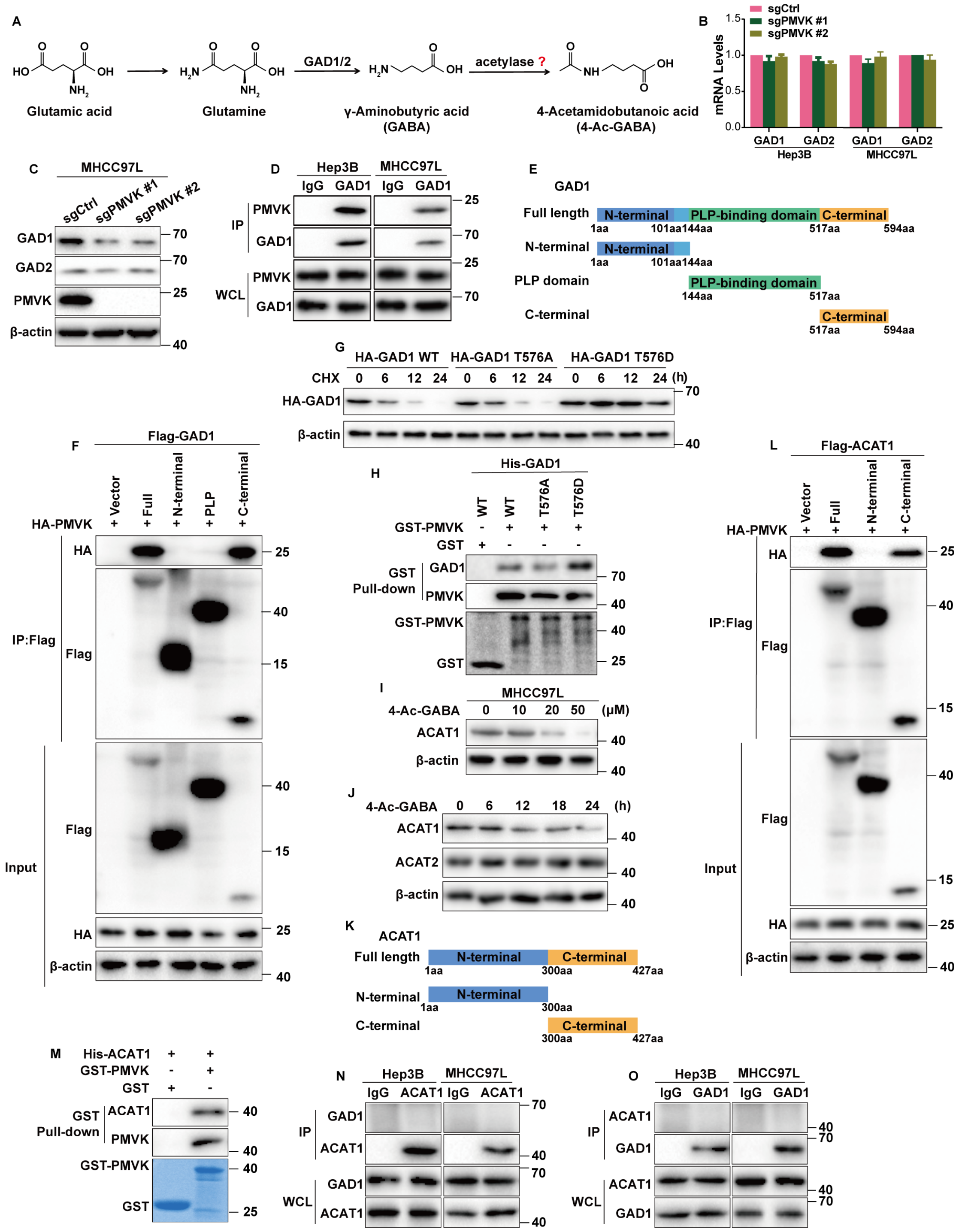

Extended Data Figure 3. PMVK recruits GAD1 and ACAT1 and increases 4-acetylaminobutyric acid levels.

(**A**) Schematic representation of GABA-related signaling pathways. (**B**) RT-PCR

analysis of GAD1 and GAD2 genes in the indicated cells, the mRNA level of ACTB were used as a reference, n = 3. Data are shown as mean ± SD. (**C**) GAD1, GAD2 and PMVK protein expression in MHCC97L cell lines. (**D**) Interaction between endogenous PMVK and GAD1 in Hep3B and MHCC97L cell lines. Anti-GAD1 Antibody from mouse was used as an IP antibody. Mouse lgG was used as a negative control. WCL, whole cell lysate. (**E**) Schematic diagram showing the structure of GAD1 and truncation mutants used. (**F**) Flag-tagged GAD1 WT or truncation mutants were co-expressed with HA-PMVK in HEK293T. Extracts were immuno-precipitated with anti-Flag antibody and examined by western blotting. Co-IP with anti-Flag antibody and then immuno-blotted with the indicated antibodies. (**G**) HEK293 cells were transfected with the indicated vectors. Cells were treated with CHX (50 μg/mL) for the indicated times and the levels of different GAD1 mutants were analyzed by western blotting. (**H**) GST pull-down assays were performed with the indicated GST-PMVK and His-GAD1 WT and its mutant fusion proteins. (**I**) MHCC97L cells were treated with 4-Ac-GABA at different concentrations and western blotting was performed with the indicated antibodies. β-actin was used as the loading control. (**J**) Hep3B cells were treated with 20μM 4-Ac-GABA at different times and western blotting was performed with the indicated antibodies. β-actin was used as the loading control. (**K**) Schematic diagram showing the structure of ACAT1 and truncation mutants used. (**L**) Flag-tagged ACAT1 WT or truncation mutants were co-expressed with HA-PMVK in HEK293T cells. Extracts were immuno-precipitated with anti-Flag antibody and examined by western blotting. (**M**) GST pull-down assays were performed with the indicated GST-PMVK

and His-ACAT1 proteins. (**N**) Interaction between endogenous ACAT1 and GAD1 in Hep3B and MHCC97L cell lines. Anti-ACAT1 Antibody from mouse was used as an IP antibody. Mouse lgG was used as a negative control. WCL, whole cell lysate. (**O**) Interaction between endogenous ACAT1 and GAD1 in Hep3B and MHCC97L cell lines. Anti-GAD1 Antibody from mouse was used as an IP antibody. Mouse lgG was used as a negative control. WCL, whole cell lysate.

Extended Data Figure 4

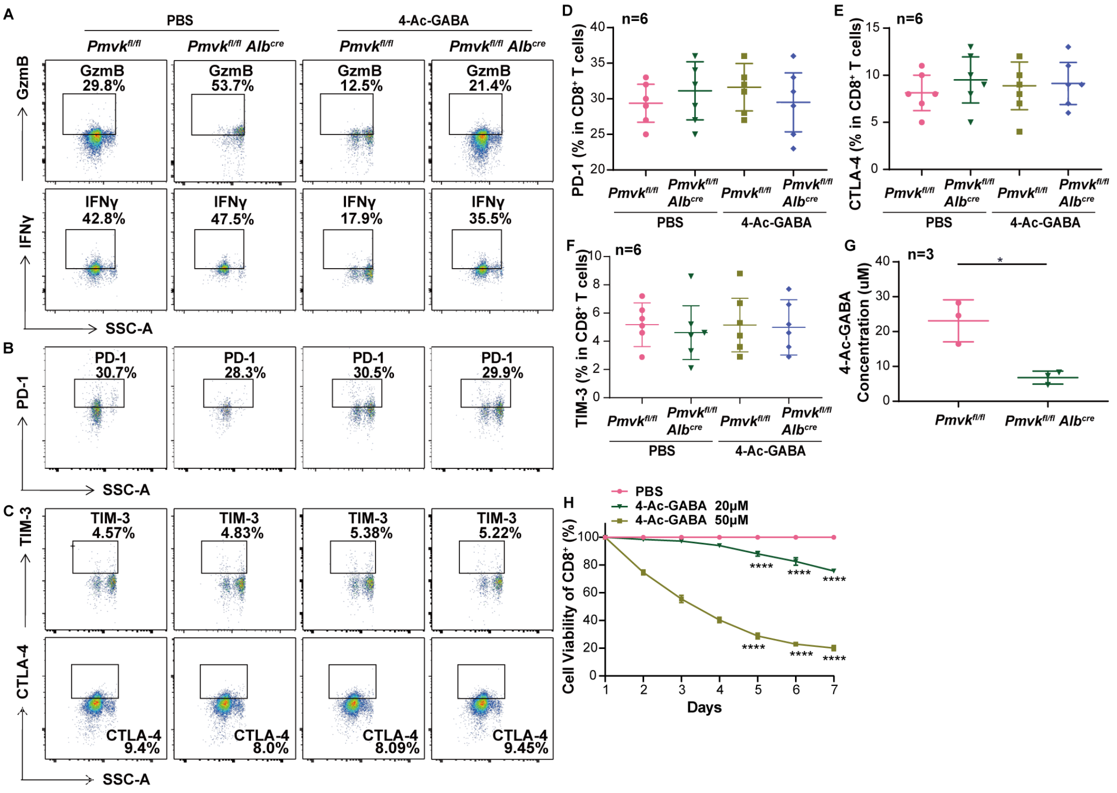

Extended Data Figure 4. 4-Ac-GABA inhibits CD8+ T cell infiltration and activation and allows HCC immune escape.

(**A-C**) Flow cytometry showing percentages of CD8+ T cells expressing GZMB, IFNγ (**A**), PD-1 (**B**), TIM-3 and CTLA-4 (**C**) from xenografts from Figure 5A. n = 6 mice per group. (**D-F**) Percentages of CD8+ T cells expressing PD-1 (**D**), TIM-3 (**E**) and CTLA-4 (**F**) in subcutaneous xenograft from Figure 5A. n = 6 mice per group. (**G**) The levels of 4-Ac-GABA in DEN/CCl_4 induced HCC tissues from PMVK hepatocyte-specific CKO mice, as measured by UPLC-MS/MS. n = 3 samples per group. (**H**) MTT assays performed on control or 4-Ac-GABA-treated splenic CD8+ T cells. Data are presented as the mean ± SD. *p < 0.05, ****p < 0.0001.

Extended Data Figure 5

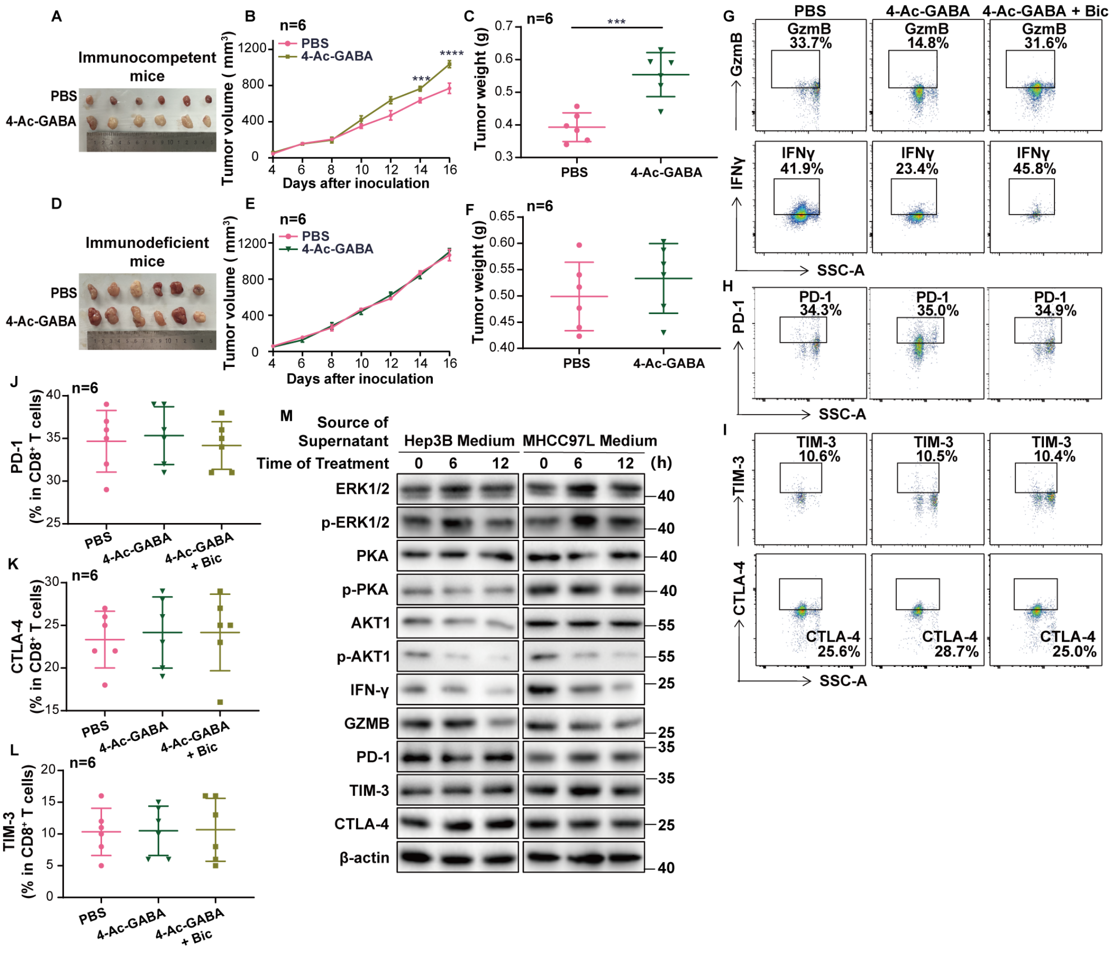

Extended Data Figure 5. Blocking the 4-acetaminobutyric acid receptor abolishes CD8⁺ T cell infiltration and HCC immune escape.

(**A**) Hepa1-6 xenografts isolated from immunocompetent mice treated with PBS or 4-Ac-GABA. n = 6 mice per group. (**B**) Xenograft tumor volumes from (**A**). (**C**) Xenograft tumor weights from (**A**). (**D**) Hepa1-6 xenografts from immunodeficient mice treated with PBS or 4-Ac-GABA. n = 6 mice per group. (**E**) Xenograft volumes from (**D**). (**F**) Subcutaneous xenograft tumor weight from (**D**). (**G-I**) Flow cytometry showing percentages $CD8^+$ T cells expressing of GZMB, IFNγ (**G**), PD-1 (**H**), TIM-3 and CTLA-4 (**I**). $CD8^+$ T cells were isolated from immunocompetent mice treated with

PBS, 4-Ac-GABA or both 4-Ac-GABA and bicuculline. n = 6 mice per group from figure 6d. (**J-L**) percentages of xenograft-derived $CD8^+$ T cells expressing PD-1 (**J**), TIM-3 (**K**) and CTLA-4 (**L**) from figure (**6D**). (**M**) Splenic $CD8^+$ T cells exposed to different media were used for western blotting. Data were presented as the mean ± SD. ***$p < 0.001$, ****$p < 0.0001$.

Extended Data Figure 6

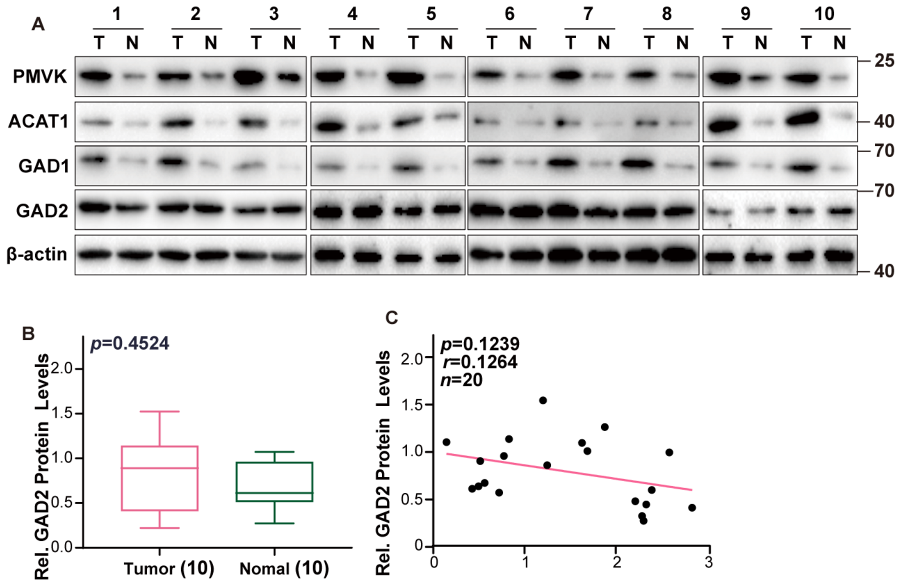

Extended Data Figure 6. PMVK is overexpressed in human HCCs.

(A) Western blot analysis for the indicated proteins in human HCCs. T, tumor; N, adjacent normal tissue. (**B**) Densitometric quantification of GAD2 for (**A**). β-actin was used as a normalizer. Data are shown as mean ± SD. (**C**) Correlation of PMVK and GAD2 protein levels in HCC tissues from (**A**). Each point is an individual sample.

Supplementary Table 3. sgRNA sequences.

sgRNA	Target Sequences (5'>3')
sgPMVK#1	TTCGGAAGGACATGATCCGC
sgPMVK#2	TGAGCATACTGTTCCTTGAG
sg*Pmvk*#1	GCGTCTGGTGCTGCTGTTTA
sg*Pmvk*#2	CTCAAGGAGGAGTATGCTCG
sg*Gad1*#1	TCTGCTCCAGAGACTCGGGG
sg*Gad1*#2	AGAAGTGAAGACAAAAGGCA
sg*Acat1*#1	AATGCCAGCACACTGAACGA
sg*Acat1*#2	GTCCCATACGTAATGAGCAG

Supplementary Table 4. Primers used for constructs.

Insert	Forward Primer (5'>3')	Reverse Primer (5'>3')
FLAG-PMVK	CGCGCGGCCGCATGGCCCCGCTGGGAGGC	CGCGTCGACAAGTCTGGAGCGGATAAATTCTATC
HA-PMVK	CGCGAATTCGGATGGCCCCGCTGGGAGGC	CGCCTCGAGCTAAAGTCTGGAGCGGATAAATTC
pET28-PMVK-His	CGCGGATCCATGGCCCCGCTGGGAGGC	CGCGAATTCTCAGTGGTGGTGGTGGTGGTGAAGTCTGGAGCGGATAAATTC
FLAG-GAD1	CGCGGATCCATGGCGTCTTCGACCCCATCTTC	CCCAAGCTTCAGATCCTGGCCCAGTCTTTCTATC
HA-GAD1	CCGGAATTCGGATGGCGTCTTCGACCCCATCTTC	CCGCTCGAGTTACAGATCCTGGCCCAGTCTTTC
pET28-GAD1-His	GATCTGGTTCCGCGTGGATCCATGGCGTCTTCGACCCCATCTTCG	ATGATGATGCGGCCGCTCGAGCAGATCCTGGCCCAGTCTTTCTA
FLAG-GAD1-N-terminal	CGCGCGGCCGC ATGGCGTCTTCGACCCCATC	CGCGGATCCTGGGTGATGAAAGTCCAGCACC
FLAG-GAD1-PLP-domain	CGCGCGGCCGCATGCACCAGTTGCTGGAAGG	CGCGGATCCATACCAAAAACAGACGTTTGTG

FLAG-GAD1-C-terminal	CGCGCGGCCGCATG-ATTCCACAAAGCCTCAGG	CGCGGATCCCAGATCCTGGCCCAGTCTTTC
FLAG-GAD1-T576A	CGCGCGGCCGCCCAGCCGCTGCCCAGTCTGAC	CGCGGATCCGTCAGACTGGGCAGCGGCTGG
HA-GAD1-T576D	CGCGGATCCCCAGCCGCTGACCAGTCTGAC	CGCGCGGCCGCGTCAGACCTGGTAGCGGCTGG
HA-GAD1-T576A	CGCGGATCCCCAGCCGCTGCCCAGTCTGAC	CGCGCGGCCGCGTCAGACTGGGCAGCGGCTGG
HA-GAD1-T551/552A	CGCGGATCCAGTCAGGTGCGGCCATGGTTG	CGCGCGGCCGCCAACCATGGCCGCACCTGACT
FLAG-ACAT1	CGCGCGGCCGCATGGCTGTGCTGGCGGCAC	CGCGGATCCCAGCTTCTGAATTAGCATGGCAG
HA-ACAT1	CGCGGATCC-ATGGCTGTGCTGGCGGCAC	CGCGCGGCCGC-CTACAGCTTCTGAATTAGCATGGCAG
FLAG-ACAT1-N-terminal	CGCGCGGCCGCATGGCTGTGCTGGCGGCAC	CGCGGATCCTGCATCTGCCGTCATGAGAACC
FLAG-ACAT1-C-terminal	CGCGCGGCCGCATGGCGAAGAGGCTCAATGTTAC	CGCGGATCCCAGCTTCTGAATTAGCATGGCAG

Supplementary Table 5. Chemical reagents and kits.

Reagent or kit	Source	Identifier
Cycloheximide	MCE	HY-12320
Methyl-β-cyclodextrin	MCE	HY-101461
PMVKi5	ChemDiv	K087-0251
DEN	Sigma-Aldrich	N0258
Trizol	Invitrogen	10296010
SYBR Green master mix	Abclonal	RK21203
MTT	Sigma-Aldrich	M5655
HiScript III 1st Strand cDNA Synthesis Kit	Vazyme	R312-01
M1P	MCE	HY-16304
G6P	MCE	HY-112537
4-Ac-GABA	MCE	HY-101411
GABA	Sigma-Aldrich	A2129
Bicuculline	MCE	HY-N0219
Propofol	MCE	HY-B0649
ECAR	Agilent Technologies	103344-100
OCR	Agilent Technologies	103015-100
Polyethylenimine	Sigma-Aldrich	49553-93-7

Puromycin Dihydrochloride	Beyotime Biotechnology	ST551
Human $CD8^+$ T Cell Isolation Kit	STEMCELL	19853A
Purified Rat Anti-Mouse CD16/CD32 (Mouse BD Fc Block)(2.4G2)	MULTI SCIENCES	70-F210163200-100
Leukocyte Activation Cocktail, with BD GolgiPlug	BD Biosciences	550583
Fixation/Permeablization Kit	MULTI SCIENCES	GAS003/2
Zombie Aqua™ Fixable Viability Kit	Biolegend	423102

Supplementary Table 6. Antibodies used in this study.

Antibody	Source	Identifier
PMVK	Proteintech	15674-1-AP
FLAG	Proteintech	25043-1-AP
HA	Abclonal	AE008
β-actin	Proteintech	66009-1-Ig
His-tag	Proteintech	66005-1-Ig
ACAT1	abclonal	A13273
GAD1	abclonal	A1475
GAD2	abclonal	A22728
AKT1	abclonal	A17909
p-AKT1-S473	abclonal	AP1208
ERK1/2	Proteintech	11257-1-AP
p-ERK-T202/Y204	Proteintech	28733-1-AP
PKA	Proteintech	55382-1-AP
P-PKA C-T197	Cell Signaling Technology	4781
PD-1	abclonal	A23007
CTLA-4	abclonal	A2063
TIM3	abclonal	A13445
IFNγ	abclonal	A12450

Granzyme B	abclonal	A2557
PE-CF594e CD279	BD Pharmingen	562523
PerCP-Cy5.5 IFN-γ	BD Pharmingen	560660
PE/Cyanine7 CD152	Biolegend	106314
APC-Cy7 CD45	BD Pharmingen	557659
BV421 CD3e	BD Pharmingen	562600
APC CD4	Biolegend	100411
BV605 CD8a	BD Pharmingen	563152
BV650 CD366	BD Pharmingen	747623
BV510 CD510	BD Pharmingen	564406
PE GRANZYME B	Biolegend	372207
Anti-mouse-CD8α-InVivo	Selleck	A2102
Anti-mouse-PD-1-InVivo	Selleck	A2122
RatlgG2b isotype control-InVivo	Selleck	A2116
ABflo™ 488-conjugated Goat Anti-Rabbit IgG	Abclonal	AS053
Goat Anti-Rabbit IgG (H+L)	Jackson	JAC-111-035-003

Goat Anti-Mouse IgG (H+L)	Jackson	JAC-115-035-003
Goat Anti-Mouse IgG Heavy Chain	Abclonal	AS064
Goat Anti-Mouse IgG Light Chain	Abclonal	AS062

Supplemental Table 7. Mouse genotypes primers.

Gene	Forward Primer (5'>3')	Reverse Primer (5'>3')
Pmvk$^{fl/fl}$	GGGAGCACCTATTTCATAGCACA	CATCATCTGCCCTACCGTCACAC
Alb-cre	CCTGTTACGTATAGCCGAAA	CTTAGCGCCGTAAATCAATC

Supplemental Table 8. qPCR primers.

Gene	Forward Primer (5'>3')	Reverse Primer (5'>3')
PMVK	CCTTTCGGAAGGACATGATCC	TCTCCGTGTGTCACTCACCA
ACTB	ATCATGAAGTGTGACGTGGACAT	AGGAGCAATGATCTTGATCTTCA
KAT1	AAGCCATTCGGAACCTTACTTC	AGTGCCATCTTTCATCATCCAC
KAT2A	GCAAGGCCAATGAAACCTGTA	TCCAAGTGGGATACGTGGTCA
KAT2B	CGAATCGCCGTGAAGAAAGC	CTTGCAGGCGGAGTACACT
KAT3A	CAACCCCAAAAGAGCCAAACT	CCTCGTAGAAGCTCCGACAGT
KAT3B	AGCCAAGCGGCCTAAACTC	TCACCACCATTGGTTAGTCCC
KAT5	GGGGAGATAATCGAGGGCTG	TCCAGACGTTTGTTGAAGTCAAT
KAT6A	TGAGTGGATTTTGGAGGCCAT	GCTATTCGCCCAGGATTATCAG
KAT6B	GCCTTGCCTCCTATAAGGACC	TCCACATTGCGGAGATCATTAC
KAT7	ATTCTGGACTGAGCAAAGAACAG	GTCATACTCGCTTGTCAGGTTTT
KAT8	GTCACGGTGGAGATCGGAGA	CCCTCCTGGTCGTTCACTC
ESCO1	AGAATTGGAAACACGCATGAGT	GATCTCCGGTTAAGCTGTTCATT
ESCO2	CACTGGGACGCACCCAAAA	CACTTGCCTTGTCGCAAAAG
CLOCK	TGCGAGGAACAATAGACCCAA	ATGGCCTATGTGTGCGTTGTA
NCOA1	CTCGGGGACAGTTCATCCG	CCGTGCTTGATGCCAGTGT
NCOA3	AGACGGGAGCAGGAAAGTAAA	GTAAAAGCGGTCCTAAGGAGTC
ATAT1	GGCCCAGAATCTTTCCGCTC	GATGCAAAGTGGTTCTACCTCAT
DLAT	CGGAACTCCACGAGTGACC	CCCCGCCATACCCTGTAGT

ACAT1	AAGGCAGGCAGTATTGGGTG	ACATCAGTTAGCCCGTCTTTTAC
ACAT2	GCGGACCATCATAGGTTCCTT	ACTGGCTTGTCTAACAGGATTCT
BRD4	ACCTCCAACCCTAACAAGCC	TTTCCATAGTGTCTTGAGCACC
NAA60	ATCGAGTACCCAGACTCATGG	TGTGTCAACAGAGAAGTTGGATG
NAA50	TCCAGTCAGCTACAATGACAAGT	CCTTCGGTAAGGTGCCAGAC
NAA10	GCGAGGCCAGAGGACCTAA	AGCCAATGAGGTGATATGTCCA
SAMHD1	CTGGAACTCCATCCCGACTAC	AGTAATGCGCCTGTGATTTCAT
HSP70	GCATCGAGACTATCGCTAATGAG	TGCAAGGTTAGATTTTTCTGCCT
PGK1	TGGACGTTAAAGGGAAGCGG	GCTCATAAGGACTACCGACTTGG
RUNX2	TGGTTACTGTCATGGCGGGTA	TCTCAGATCGTTGAACCTTGCTA
TAF1	CGGACAGCAGCTACCATCAC	CGTCAGTCCCGGTCAATTCT
GTF3C1	GGGAAGCTGCACTATCACAGA	GGTAATCGGATCACATGGGACT
GTF3C2	GGCTTCCGTAGAGATGTCATTAC	GCCTTTTCGTGTCCTACCACC
GTF3C4	CTCCTCAAAGTTGGCTCAAAAAC	AACATGAAAGTCTGACTGACCG
ELP3	CTGCCGTCCCTCCTCAGTAT	ACAGCCACGACAGCAATCC
BLOC1S1	AGGAGGCGAGAGGCTATCAC	GGACCTGTAGGGTCTTCACCT
ATF2	AATTGAGGAGCCTTCTGTTGTAG	CATCACTGGTAGTAGACTCTGGG
GANP	TCTAAGCCGCCATTTCGATTT	AAGACGCTGGAAAGCTGGATA
ATAC2	AGGGAACAGCTCAGTTACCTT	ATGACGACTTGCTGCCATGTC
MAPT	CCAAGTGTGGCTCATTAGGCA	CCAATCTTCGACTGGACTCTGT
NAT10	ATAGCAGCCACAAACATTCGC	ACACACATGCCGAAGGTATTG

Gabra1	AAAAGTCGGGGTCTCTCTGAC	CAGTCGGTCCAAAATTCTTGTGA
Gabra2	GGACCCAGTCAGGTTGGTG	TCCTGGTCTAAGCCGATTATCAT
Gabra3	ATGTGGCACTTTTATGTGACCA	CCCCAGGTTCTTGTCGTCTTG
Gabra4	AGACTCACCATAAGTGCGGAG	GCCTTTGGTCCAGGTGTAGAT
Gabra5	GCAGGTGCGAACAGACATCTA	CCTTAAACCGCAGCCTTTCATC
Gabra6	TTGCTCACAACATGACCACCC	GGGGCAATCAGCGTTGATG
Gabrb1	AGGGGCATATCCACGACTATC	ACCCAGGACAGGATTGTAATCAG
Gabrb2	ATGTCGCTGGTTAAAGAGACG	CTGCCACTCGGTTGTCCAAA
Gabrb3	CTGCTGCCAATCTGGCTTTC	CGTAGCCTTTCAACAGCTTGTC
Gabrd	ATTGGGGACTACGTGGGCT	CAAGCGCCACATTCACAGG
Gabre	CCTTCAGTGGAGGGTTGGAC	ATTCAGGCGGAGTTAGAGGCT
Gabrg1	GCGTGAGACCCACAGTGATT	TGCATCCGATTTTCTTGAGTTCC
Gabrg2	AGAAAAACCCTCTTCTTCGGATG	GTGGCATTGTTCATTTGAATGGT
Gabrg3	GACAGTCGCCTTCGATTCAAC	AGCCTCCGCTGTTTTAGAATTT
Gabrp	CAGACCCACGGCTAGTGTTC	AGAGGCGGATGAGCCTGTT
Gabrq	ATGGGCATCCGAGGTATGCT	ATCCAGAATGACTTCAGGCGT
Gabrr1	CGAGGAGCACACGACGATG	GTGAAGTCCATGTCAACCTCTG
Gabrr2	TGAGACTCGCTTTGGTCTTGT	CCACACCTACAGGGATGGC
Gabrr3	CACCCTAAACGTGAACAACTGT	TCCAATAGTGCCTGAGGTAAAAC
GAD1	GCGGACCCCAATACCACTAAC	CACAAGGCGACTCTTCTCTTC
GAD2	TTTTGGTCTTTCGGGTCGGAA	TTCTCGGCGTCTCCGTAGAG
